# Supplementary material for: Anti-Browning Effect of 2-Mercaptobenzo[d]imidazole Analogs with Antioxidant Activity on Freshly-Cut Apple Slices and Their Highly Potent Tyrosinase Inhibitory Activity
Source: Antioxidants (Basel). 2023 Sep 29;12(10):1814. doi: 10.3390/antiox12101814 (PMC10604187; doi:10.3390/antiox12101814)
Supplement: Supplementary file 1 [file antioxidants-12-01814-s001.zip › antioxidants-2633926-supplementary.pdf]

Supplementary Material

Anti-Browning Effect of 2-Mercaptobenzo[*d*]imidazole Analogs with Antioxidant Activity on Freshly-Cut Apple Slices and Their Highly Potent Tyrosinase Inhibitory Activity

Jieun Lee [1,†], Hye Soo Park [1,†], Hee Jin Jung [2,†], Yu Jung Park [1], Min Kyung Kang [1], Hye Jin Kim [1], Dahye Yoon [1], Sultan Ullah [3], Dongwan Kang [4], Yujin Park [4], Pusoon Chun [5], Hae Young Chung [2] and Hyung Ryong Moon [1,*]

[1] Department of Manufacturing Pharmacy, College of Pharmacy and Research Institute for Drug Development, Pusan National University, Busan 46241, Republic of Korea; yijiun@pusan.ac.kr (J.L.); hyesoo0713@pusan.ac.kr (H.S.P.); wjd9933@pusan.ac.kr (Y.J.P.); kmk87106@pusan.ac.kr (M.K.K.); khj3358@pusan.ac.kr (H.J.K.); dahae0528@pusan.ac.kr (D.Y.)
[2] Department of Pharmacy, College of Pharmacy and Research Institute for Drug Development, Pusan National University, Busan 46241, Republic of Korea; hjjung2046@pusan.ac.kr (H.J.J.); hyjung@pusan.ac.kr (H.Y.C.)
[3] Department of Molecular Medicine, The Herbert Wertheim UF Scripps Institute for Biomedical Innovation & Technology, Jupiter, FL 33458, USA; sultanullahf@ufl.edu
[4] Department of Medicinal Chemistry, New Drug Development Center, Daegu-Gyeongbuk Medical Innovation Foundation, Daegu 41061, Republic of Korea; kdw4106@kmedihub.re.kr (D.K.); pyj1016@kmedihub.re.kr (Y.P.)
[5] College of Pharmacy and Inje Institute of Pharmaceutical Sciences and Research, Inje University, Gimhae 50834, Republic of Korea; pusoon@inje.ac.kr
* Correspondence: mhr108@pusan.ac.kr; Tel.: +82-51-510-2815; Fax: +82-51-513-6754
† These authors contributed equally to this work.

Contents

1. Synthesis of benzimidazole derivatives without the 2-mercapto substituent of 2-MBI analogs……………………………………………………………………………….….4

Table S1…………………………………………………………………………......6

Table S2……………………………………………………………..………..…...8

Table S3……………………………………………………………..……......……10

Table S4………………………………………………..……………………….……..10

Figure S1. ^{1}H NMR spectrum of analog **1**………………………………………….………11

Figure S2. ^{13}C NMR spectrum of analog **1**…………………………………………………12

Figure S3. ^{1}H NMR spectrum of analog **2**…………………………………………………13

Figure S4. ^{13}C NMR spectrum of analog **2**…………………………………………………14

Figure S5. ^{1}H NMR spectrum of analog **3**…………………………………………………15

Figure S6. ^{13}C NMR spectrum of analog **3**………………………………………………...16

Figure S7. ^{1}H NMR spectrum of analog **4**…………………………………………………17

Figure S8. ^{13}C NMR spectrum of analog **4**………………………………………………...18

Figure S9. ^{1}H NMR spectrum of analog **5**…………………………………………………19

Figure S10. ^{13}C NMR spectrum of analog **5**……………………………………………….20

Figure S11. ^{1}H NMR spectrum of analog **6**……………………………………………...21

Figure S12. ^{13}C NMR spectrum of analog **6**……………………………………………….22

Figure S13. ^{1}H NMR spectrum of analog **7**……………………………………………………23

Figure S14. ^{13}C NMR spectrum of analog **7**……………………………………………………24

Figure S15. ^{1}H NMR spectrum of analog **8**……………………………………………………25

Figure S16. ^{13}C NMR spectrum of analog **8**…………………………………………………...26

Figure S17. ^{1}H NMR spectrum of analog **9**……………………………………………………27

Figure S18. ^{13}C NMR spectrum of analog **9**……………………………………………………28

Figure S19. ^{1}H NMR spectrum of analog **10**…………………………………………………...29

Figure S20. ^{13}C NMR spectrum of analog **10**…………………………………………………..30

Figure S21. ^{1}H NMR spectrum of analog 1*H*-benzo[*d*]imidazole…………………………...31

Figure S22. ^{13}C NMR spectrum of analog 1*H*-benzo[*d*]imidazole…………………………..32

Figure S23. ^{1}H NMR spectrum of analog 5-methyl-1*H*-benzo[*d*]imidazole………………...33

Figure S24. ^{13}C NMR spectrum of analog 5-methyl-1*H*-benzo[*d*]imidazole………………..34

Figure S25. ^{1}H NMR spectrum of analog 5-nitro-1*H*-benzo[*d*]imidazole…………………...35

Figure S26. ^{13}C NMR spectrum of analog 5-nitro-1*H*-benzo[*d*]imidazole…………………..36

Figure S27. ^{1}H NMR spectrum of analog 5-chloro-1*H*-benzo[*d*]imidazole…………………37

Figure S28. ^{13}C NMR spectrum of analog 5-chloro-1*H*-benzo[*d*]imidazole…………………38

1. Synthesis of benzimidazole derivatives without the 2-mercapto substituent of 2-MBI analogs

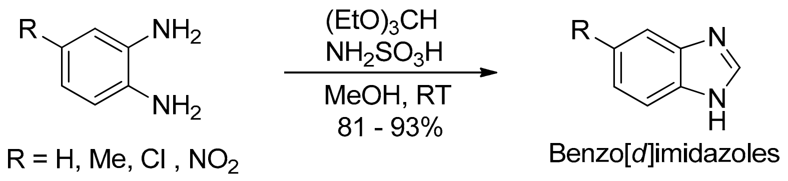

1.1. General procedures for 1,2-phenylenediamine derivatives

A solution of 1,2-phenylenediamine derivatives (1,2-phenylenediamine, 4-methyl-1,2-phenylenediamine, 4-chloro-1,2-phenylenediamine, and 4-nitro-1,2-phenylenediamine) (100 mg) and triethyl orthoformate (1.2 equiv.) in methanol (3 mL) was stirred in the presence of sulfamic acid (0.05 equiv.) at room temperature for 4 – 12 h. After cooling, the reaction mixture was partitioned between ethyl acetate and water and the organic layer was dried over $MgSO_4$, filtered, and evaporated to afford 1,2-phenylenediamine derivatives as solids in 81–93 % yields.

1.2. NMR data for benzimidazole derivatives without the 2-mercapto substituent of 2-MBI analogs

1*H*-Benzo[*d*]imidazole

^{1}H NMR (400 MHz, DMSO-*d*$_6$) δ 12.39 (brs, 1H, NH), 8.20 (s, 1H, 2-H), 7.65–7.55 (brm, 2H, 4-H, 7-H), 7.18 (dd, 2H, J = 6.0, 2.8 Hz, 5-H, 6-H); ^{13}C NMR (100 MHz, DMSO-*d*$_6$) δ 142.5, 138.7, 122.3, 115.9.

5-Methyl-1*H*-benzo[*d*]imidazole

^{1}H NMR (400 MHz, $CDCl_3$) δ 8.05 (s, 1H, 2-H), 7.56 (d, 1H, J = 8.0 Hz, 7-H), 7.44 (d, 1H, J = 1.2 Hz, 4-H), 7.12 (dd, 1H, J = 8.0, 1.2 Hz, 6-H), 2.48 (s, 3H, CH_3); ^{13}C NMR (100 MHz,

$CDCl_3$) δ 140.6, 137.5, 136.4, 132.8, 124.5, 115.5, 114.9, 21.7.

5-Nitro-1*H*-benzo[*d*]imidazole

^{1}H NMR (400 MHz, DMSO-d_6) δ 8.54 (s, 1H, 2-H), 8.51 (d, 1H, *J* = 2.4 Hz, 4-H), 8.11 (dd, 1H, *J* = 8.8, 2.4 Hz, 6-H), 7.76 (d, 1H, *J* = 8.8 Hz, 7-H); ^{13}C NMR (100 MHz, DMSO-d_6) δ 147.3, 143.2, 142.1, 139.0, 118.2, 115.5, 113.3.

5-Chloro-1*H*-benzo[*d*]imidazole

^{1}H NMR (400 MHz, $CDCl_3$) δ 8.12 (s, 1H, 2-H), 7.66 (d, 1H, *J* = 2.0, 4-H), 7.58 (d, 1H, *J* = 8.8 Hz, 7-H), 7.27 (dd, 1H, *J* = 8.8, 2.0 Hz, 6-H); ^{13}C NMR (100 MHz, $CDCl_3$) δ 141.8, 138.4, 136.5, 128.8, 123.7, 116.4, 115.4.

Table S1. Docking simulation of 2-MBI analogs, **3** – **5** and **7** – **10** and mushroom tyrosinase (PDB ID: 2Y9X) using the AutoDock Vina

Compd.	Pharmacophore analysis	3D structure	Docking score (Kcal/mol)
**3**	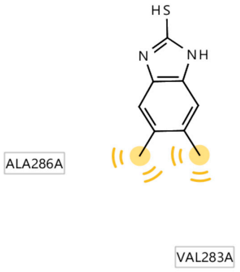	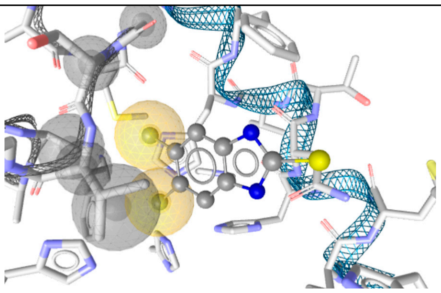	–5.9
**4**	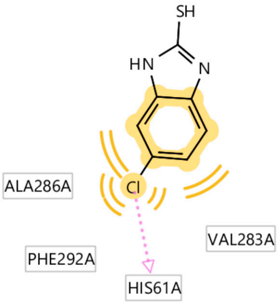	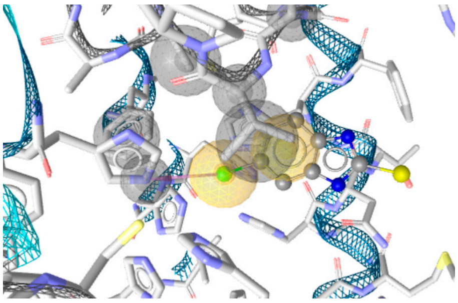	–5.6
**5**	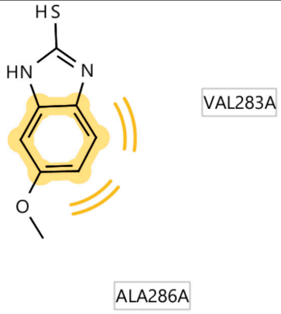	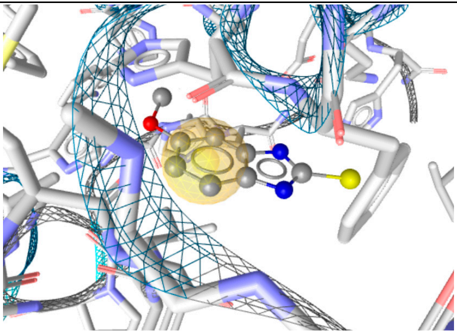	–5.9
**7**	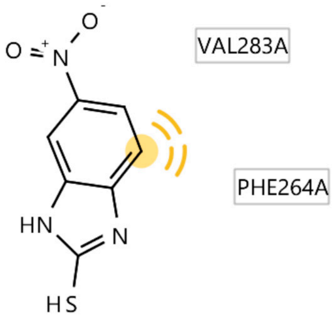	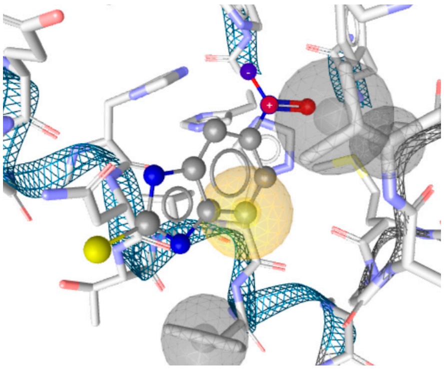	–5.8

**8**	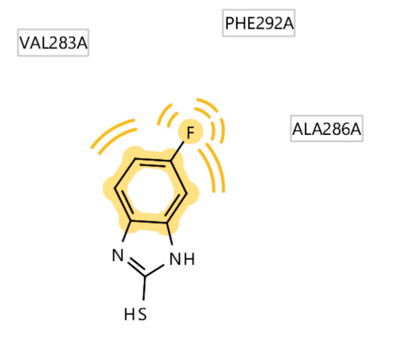VAL283A PHE292A ALA286A F N NH HS	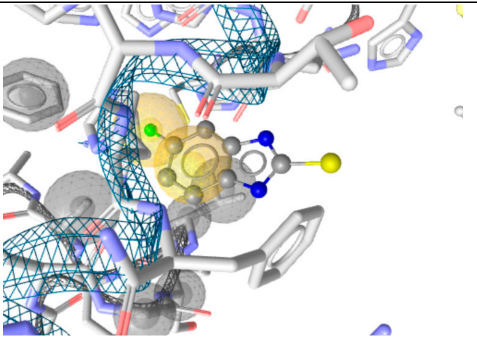	–5.9
**9**	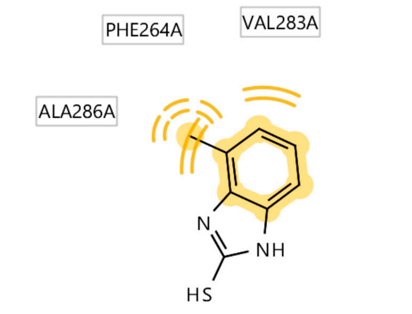PHE264A VAL283A ALA286A N NH HS	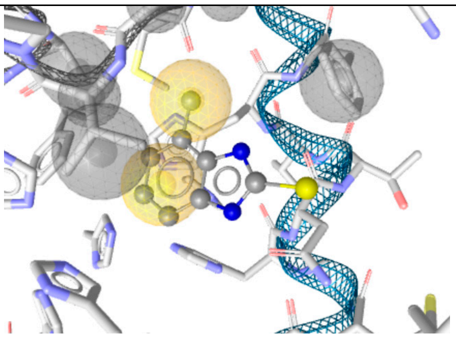	–5.8
**10**	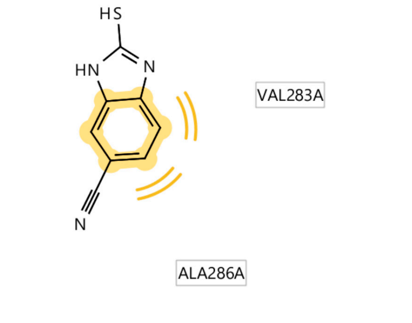HS HN N VAL283A N ALA286A	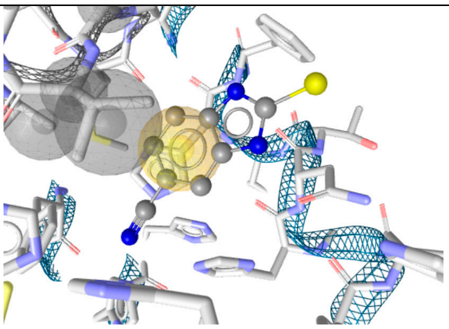	–5.9

Table S2. Docking simulation of benzo[*d*]imidazoles lacking of the 2-mercapto substituent in 2-MBI analogs **1–10** and mushroom tyrosinase (PDB ID: 2Y9X) using the AutoDock Vina.

2-Demercapto compd.	Pharmacophore analysis	3D structure	Docking score (Kcal/mol)
**1′**	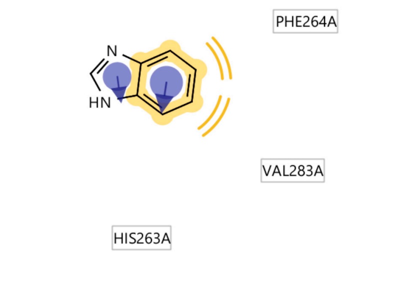	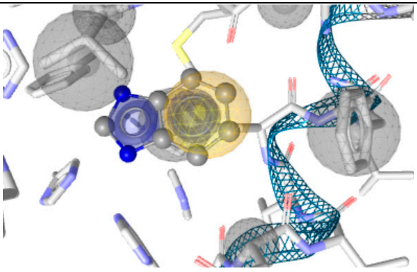	–6.0
**2′**	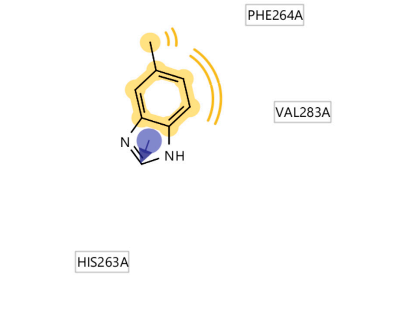	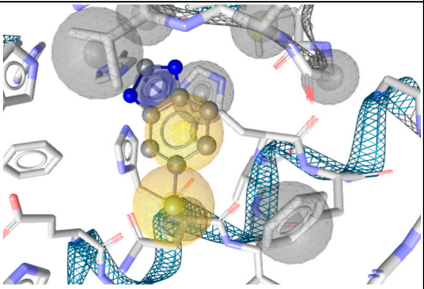	–5.9
**3′**	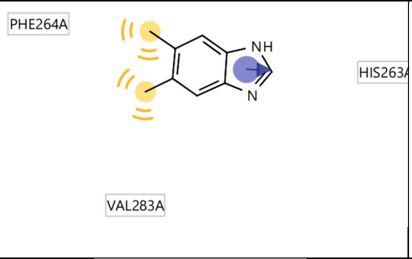	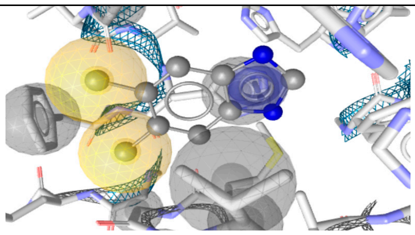	–5.9
**4′**	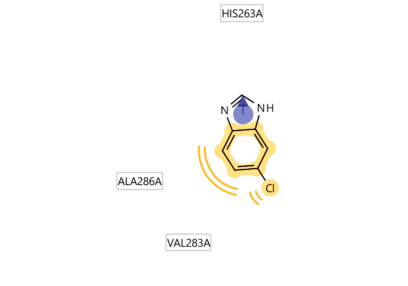	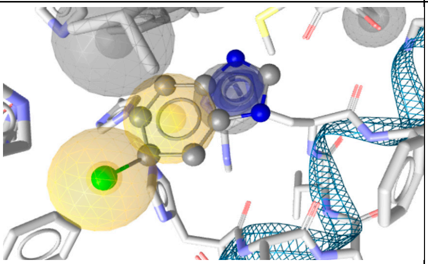	–5.9
**5′**	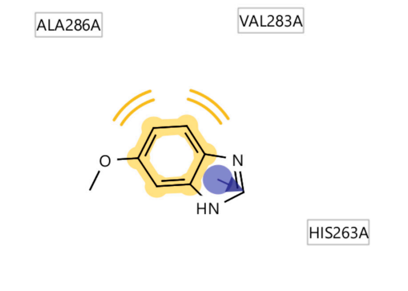	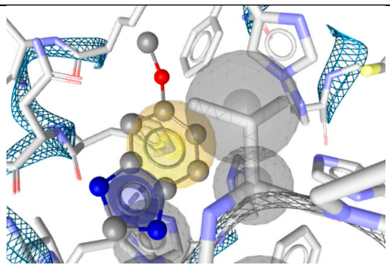	–6.0

**6′**	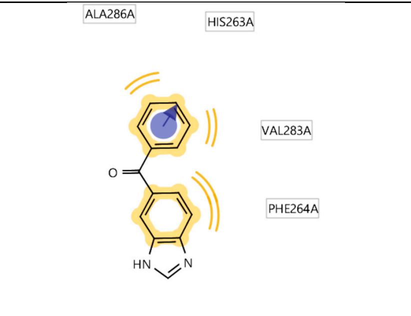	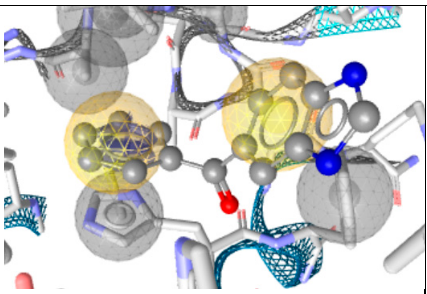	–7.5
**7′**	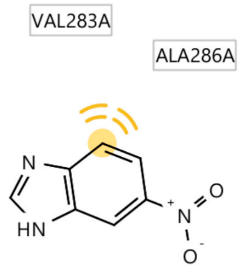	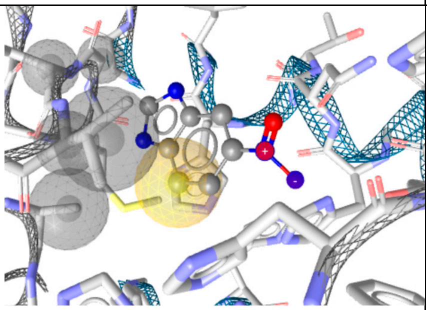	–6.1
**8′**	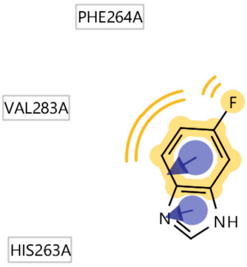	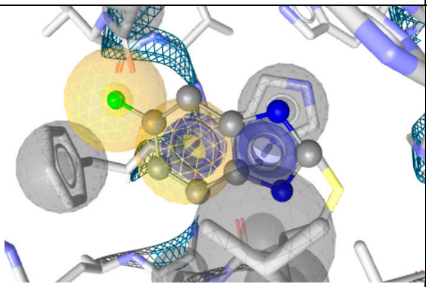	–6.3
**9′**	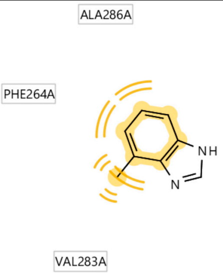	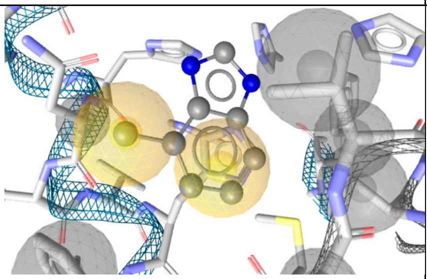	–6.0
**10′**	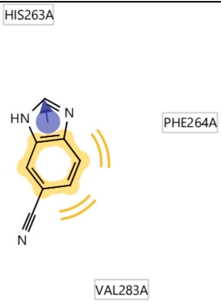	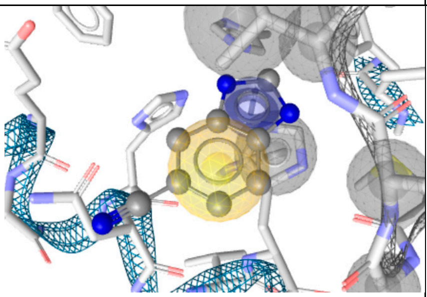	–6.2

Table S3. Mushroom tyrosinase inhibition of 2-imidazolinethione at 3 different concentrations (4, 20 and 100 µM) in the presence of L-dopa

Compound	Concentration (µM)	Tyrosinase activity inhibition (%)
2-imidazolinethione	4	3.16 ± 1.26
	20	5.34 ± 0.42
	100	5.34 ± 0.63

Table S4. Docking simulation of 2-imidazolinethione and mushroom tyrosinase (PDB ID: 2Y9X) using the AutoDock Vina

Compd.	Pharmacophore analysis	3D structure	Docking score (Kcal/mol)
2-imidazolinethione	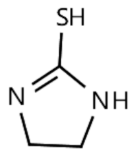 No characteristic interactions.	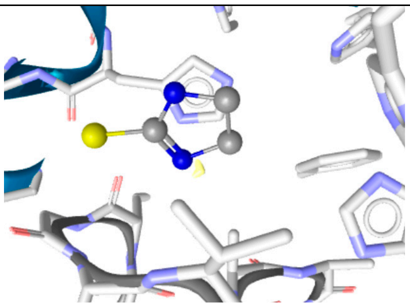	–3.6

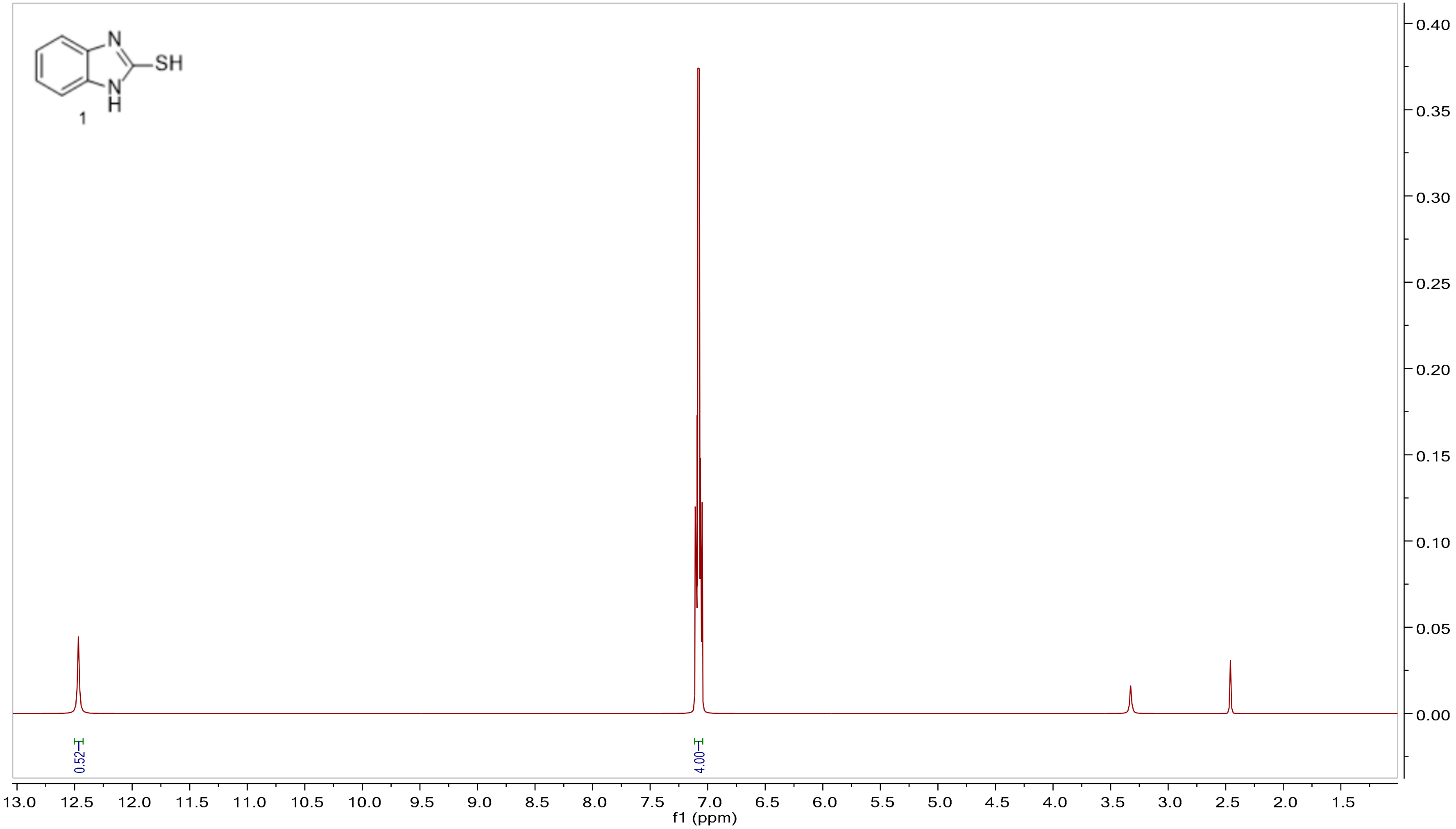

Figure S1. ^{1}H NMR spectrum of analog **1**

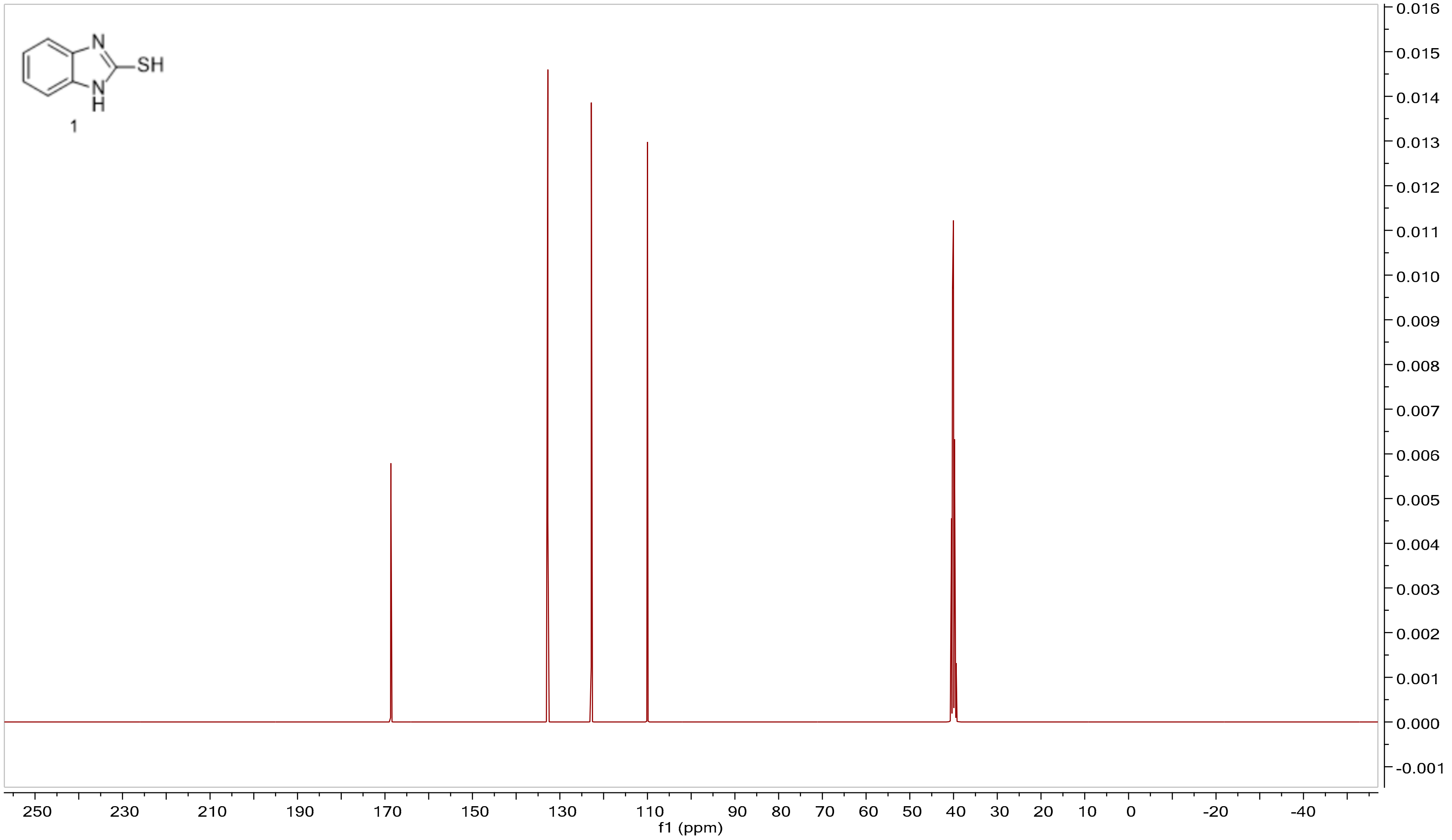

Figure S2. ^{13}C NMR spectrum of analog **1**

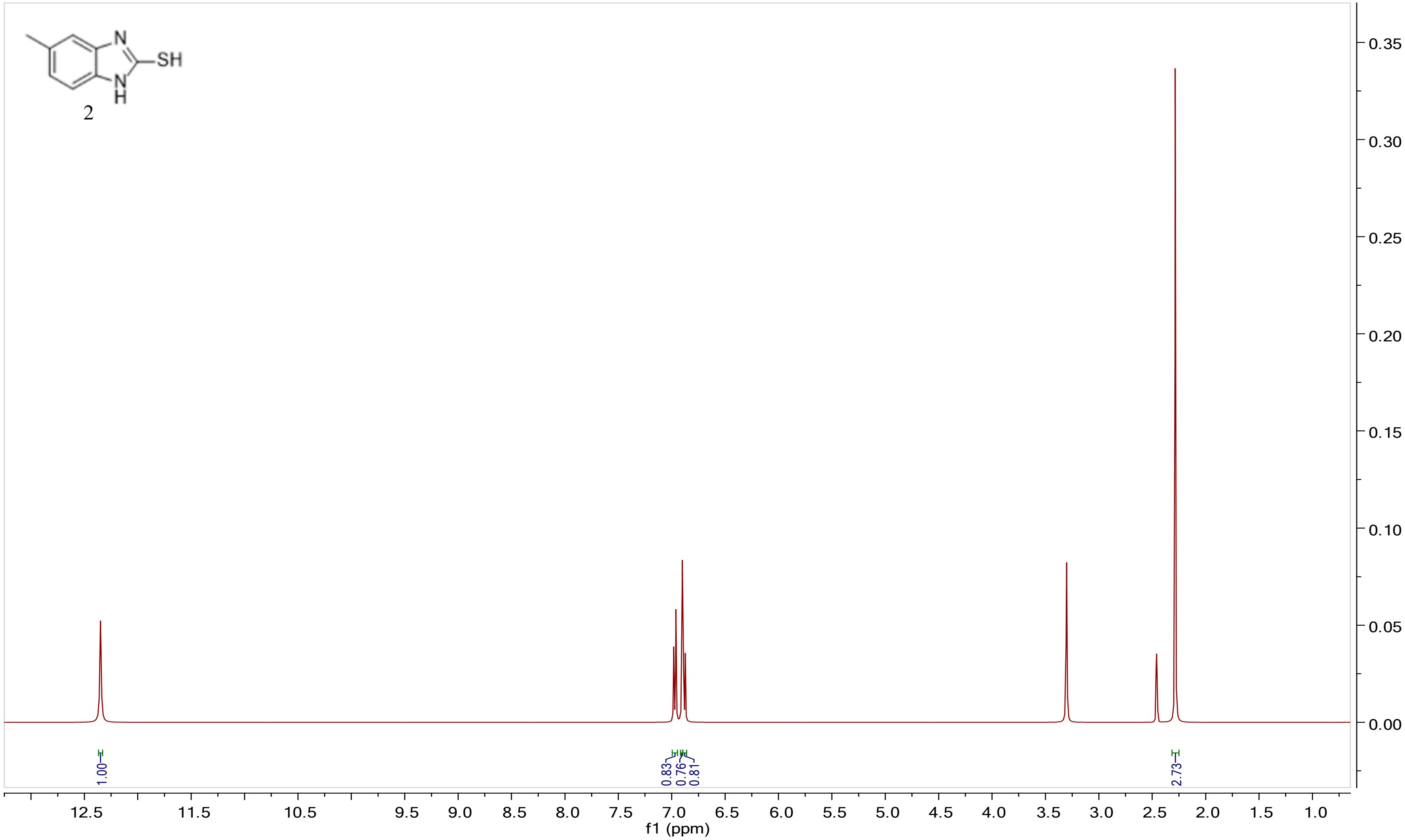

Figure S3. ^{1}H NMR spectrum of analog **2**

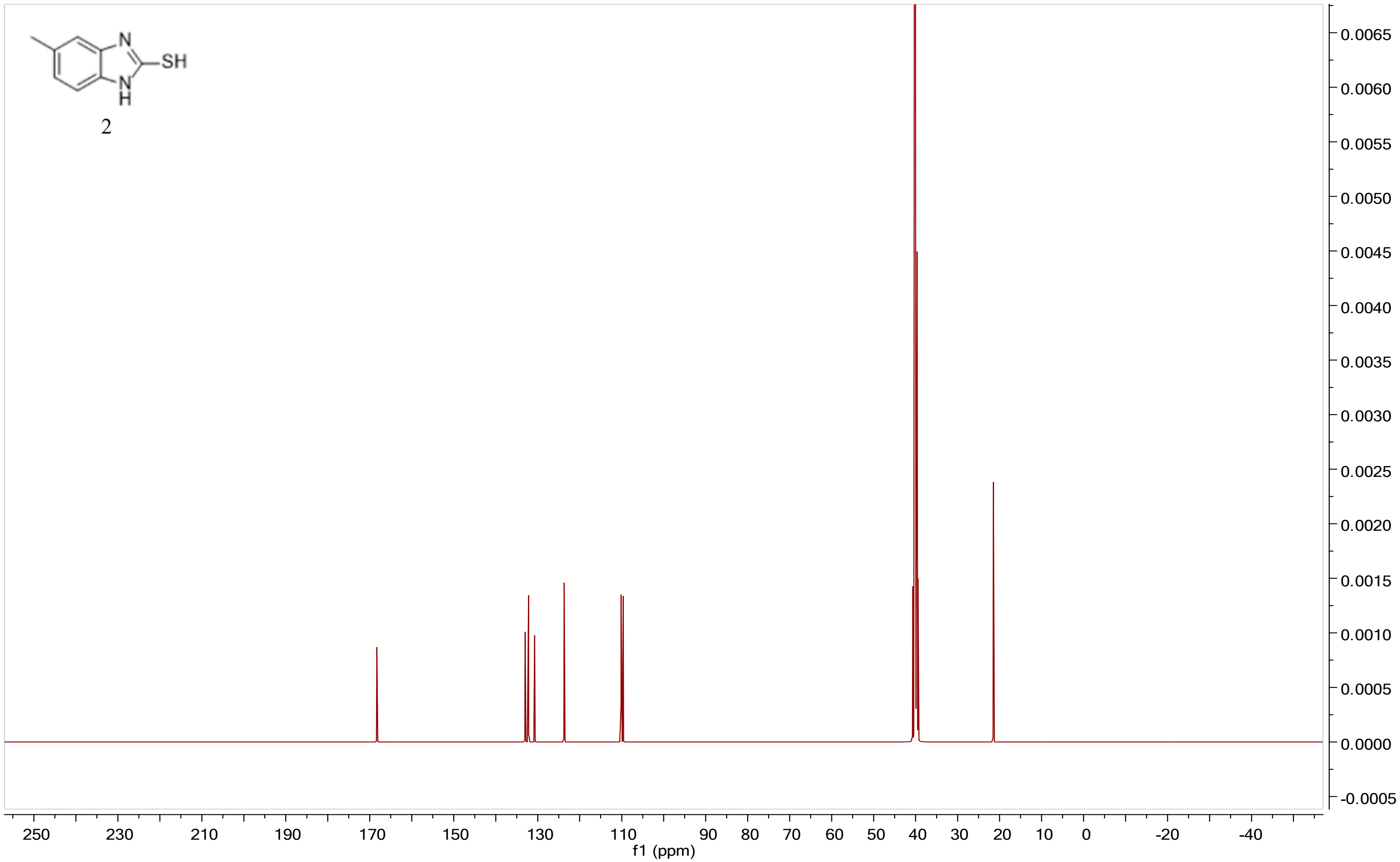

Figure S4. ^{13}C NMR spectrum of analog **2**

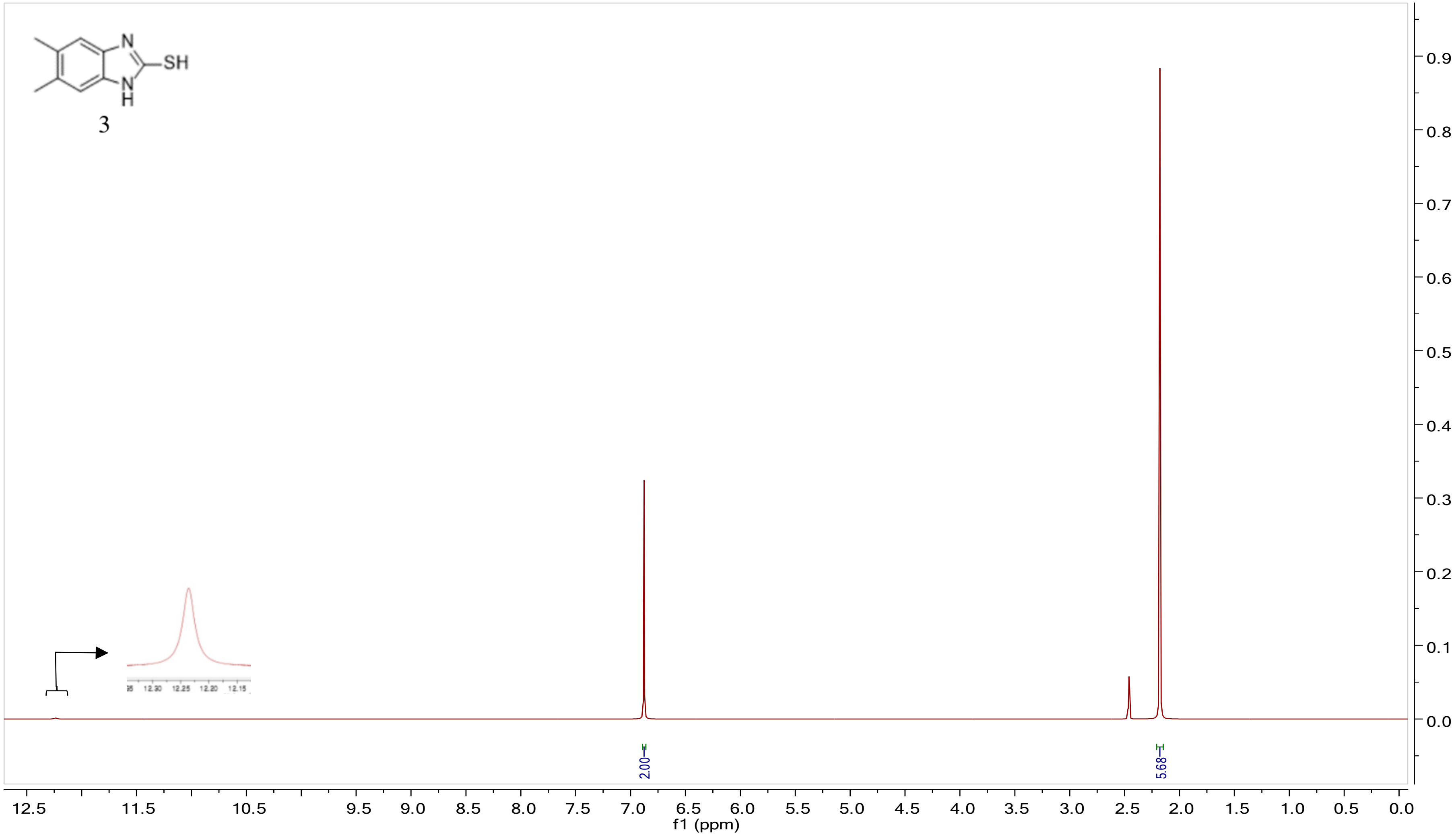

Figure S5. ^{1}H NMR spectrum of analog **3**

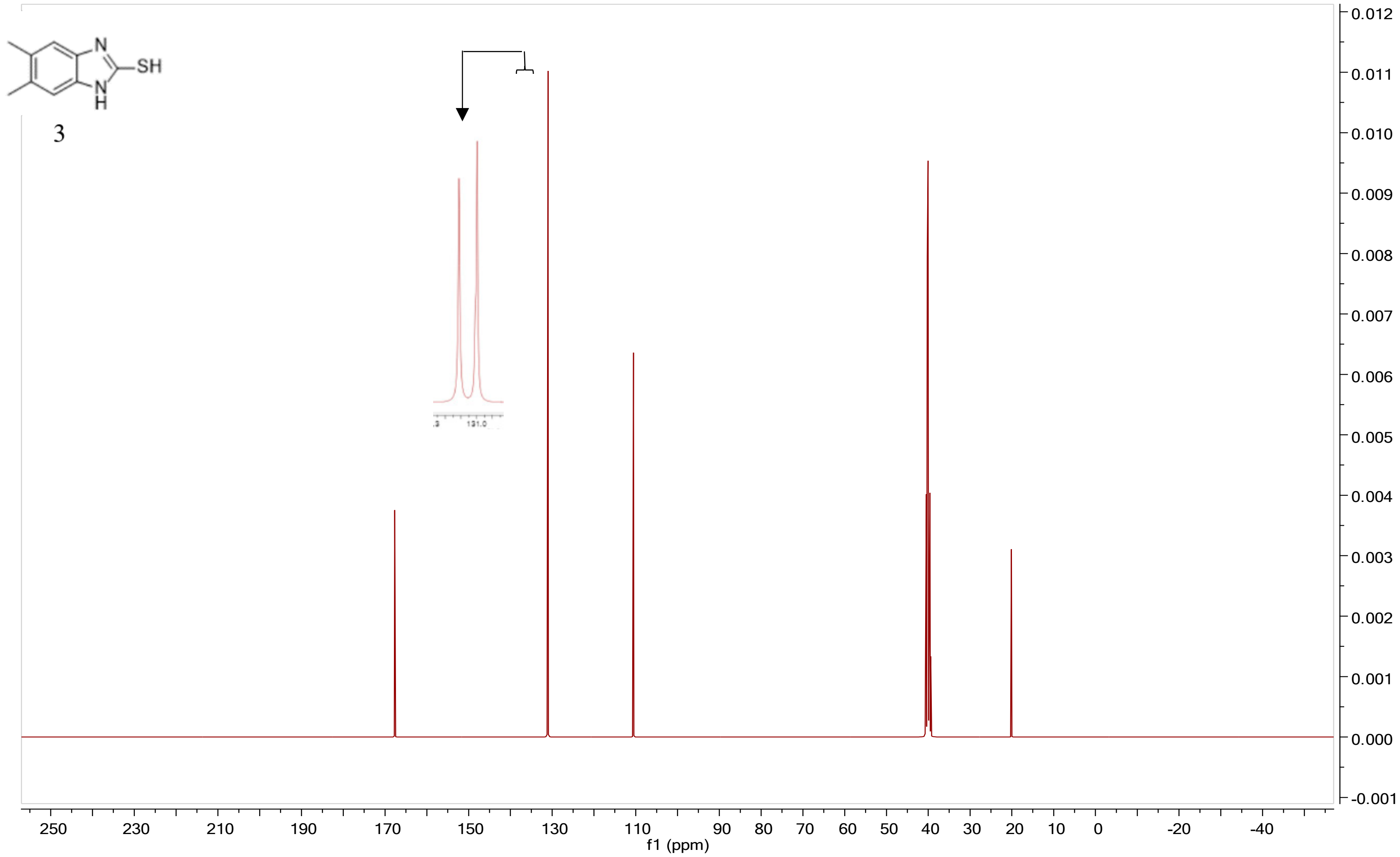

Figure S6. ^{13}C NMR spectrum of analog **3**

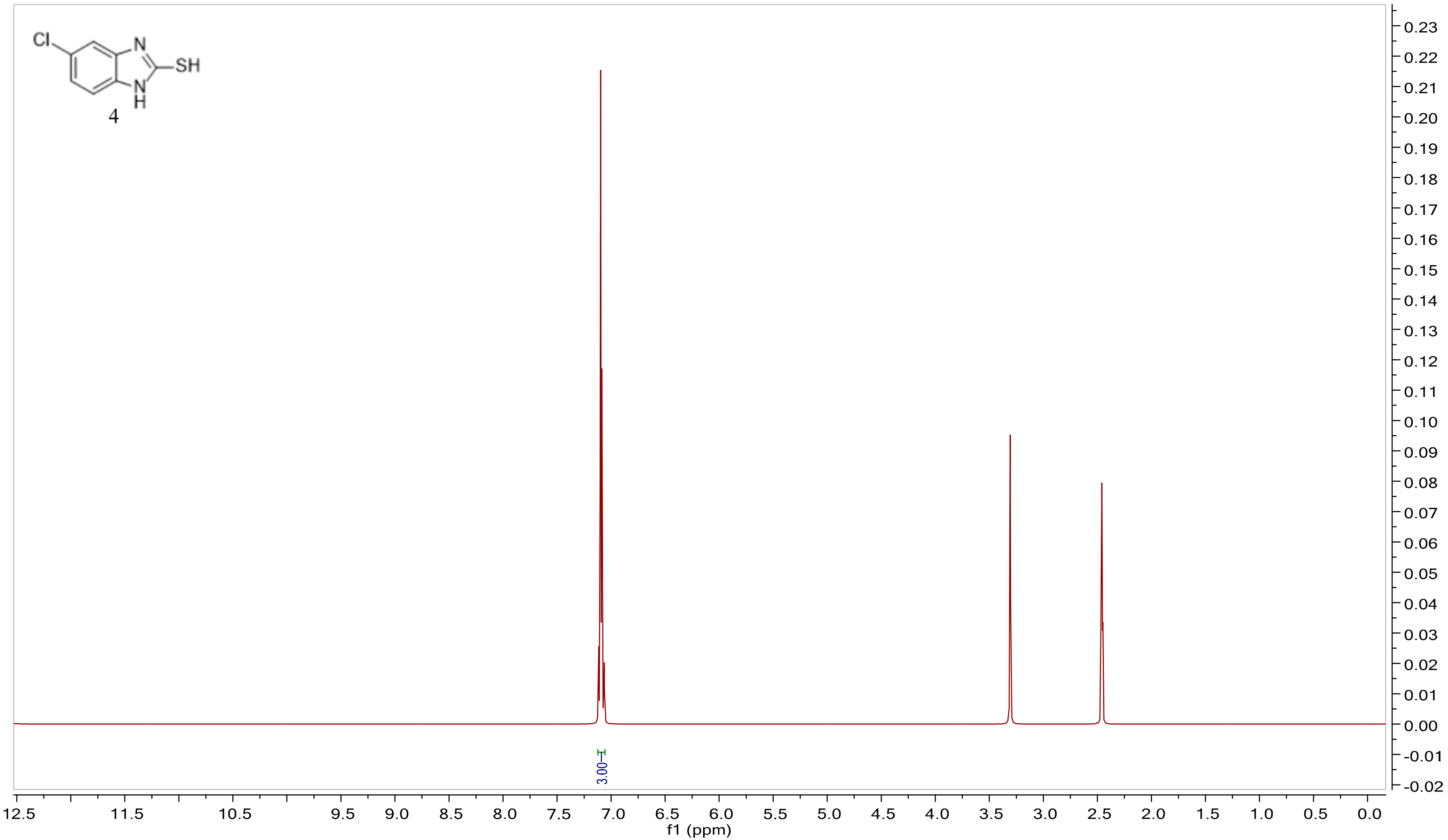

Figure S7. ^{1}H NMR spectrum of analog **4**

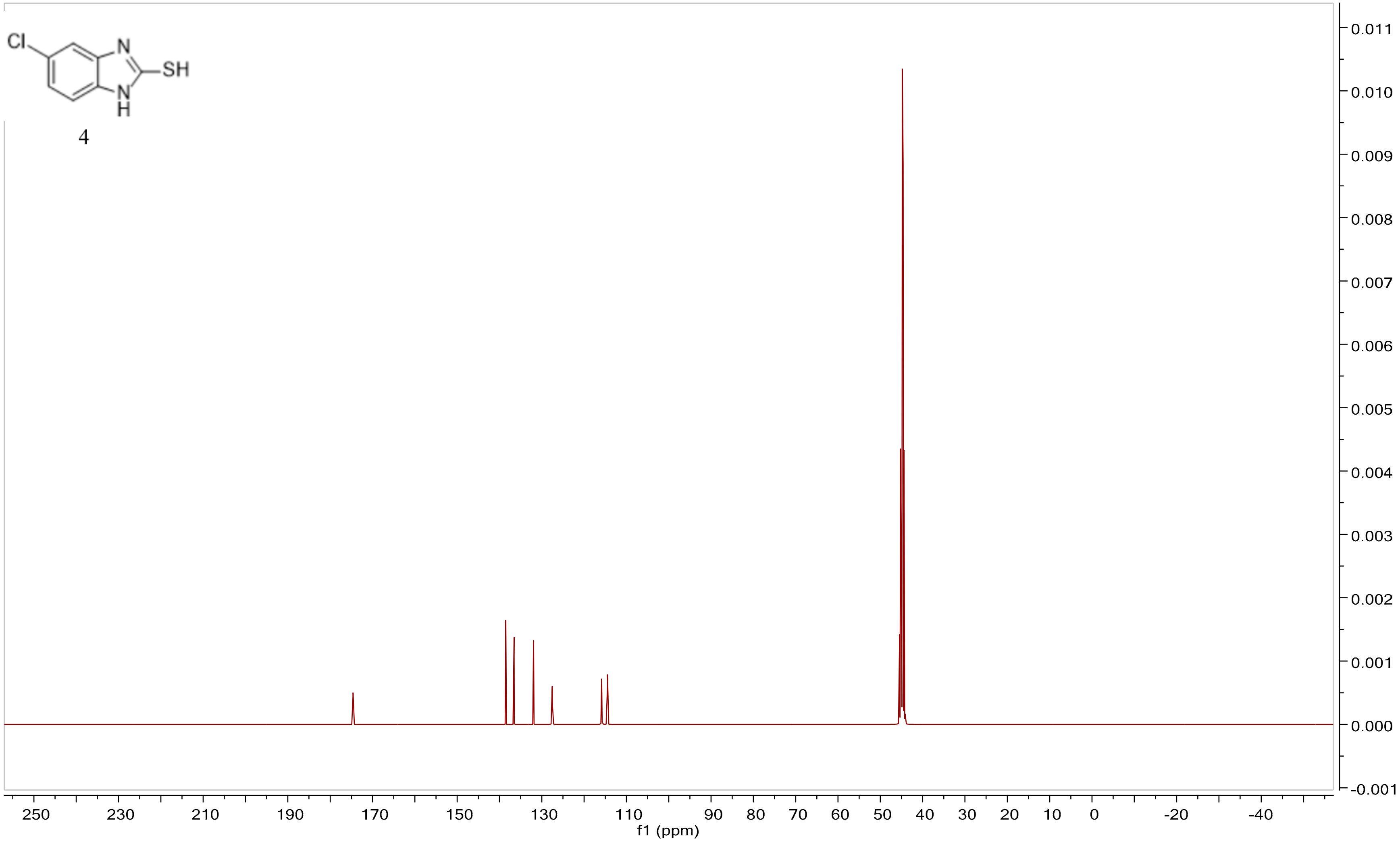

Figure S8. ^{13}C NMR spectrum of analog **4**

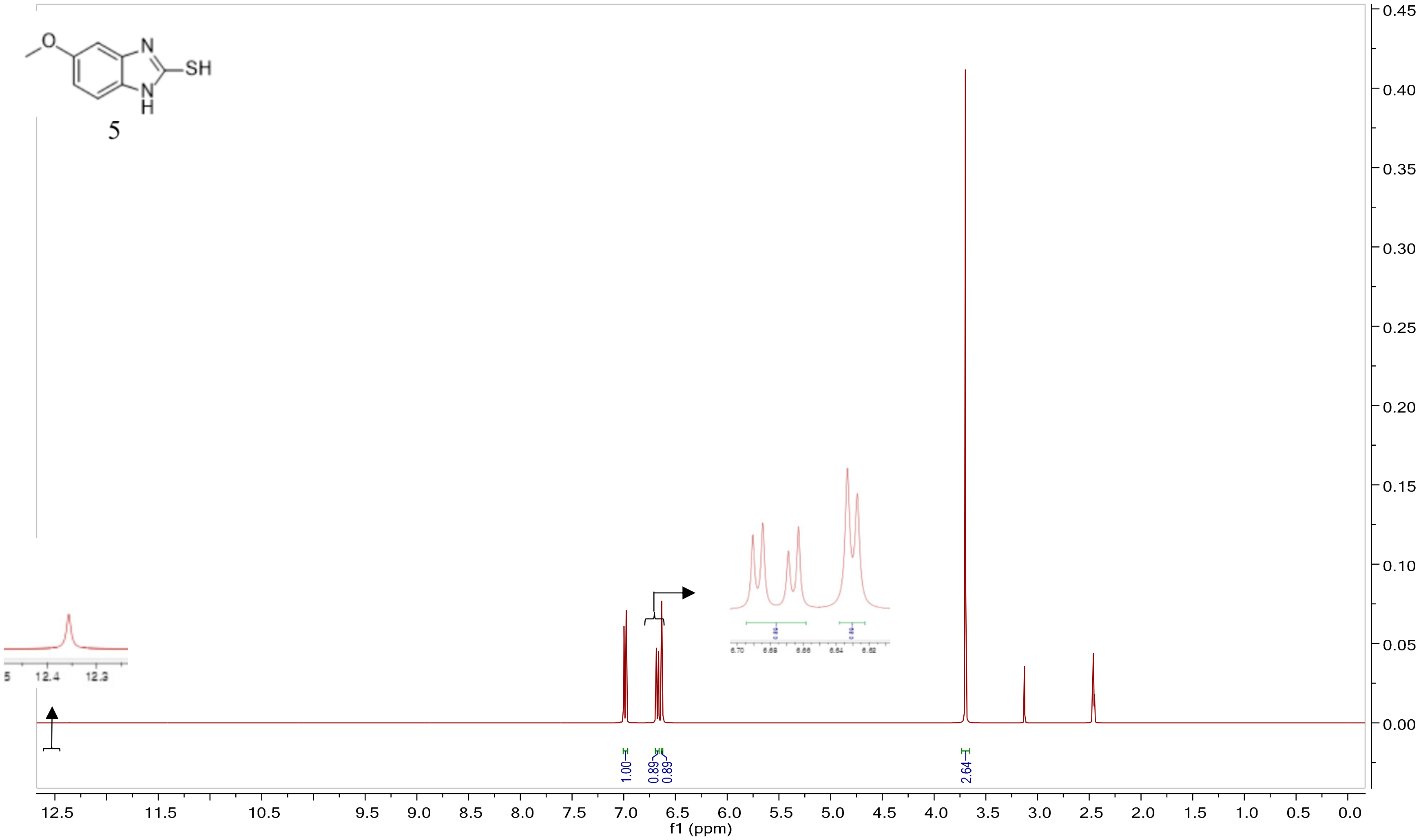

Figure S9. ^{1}H NMR spectrum of analog **5**

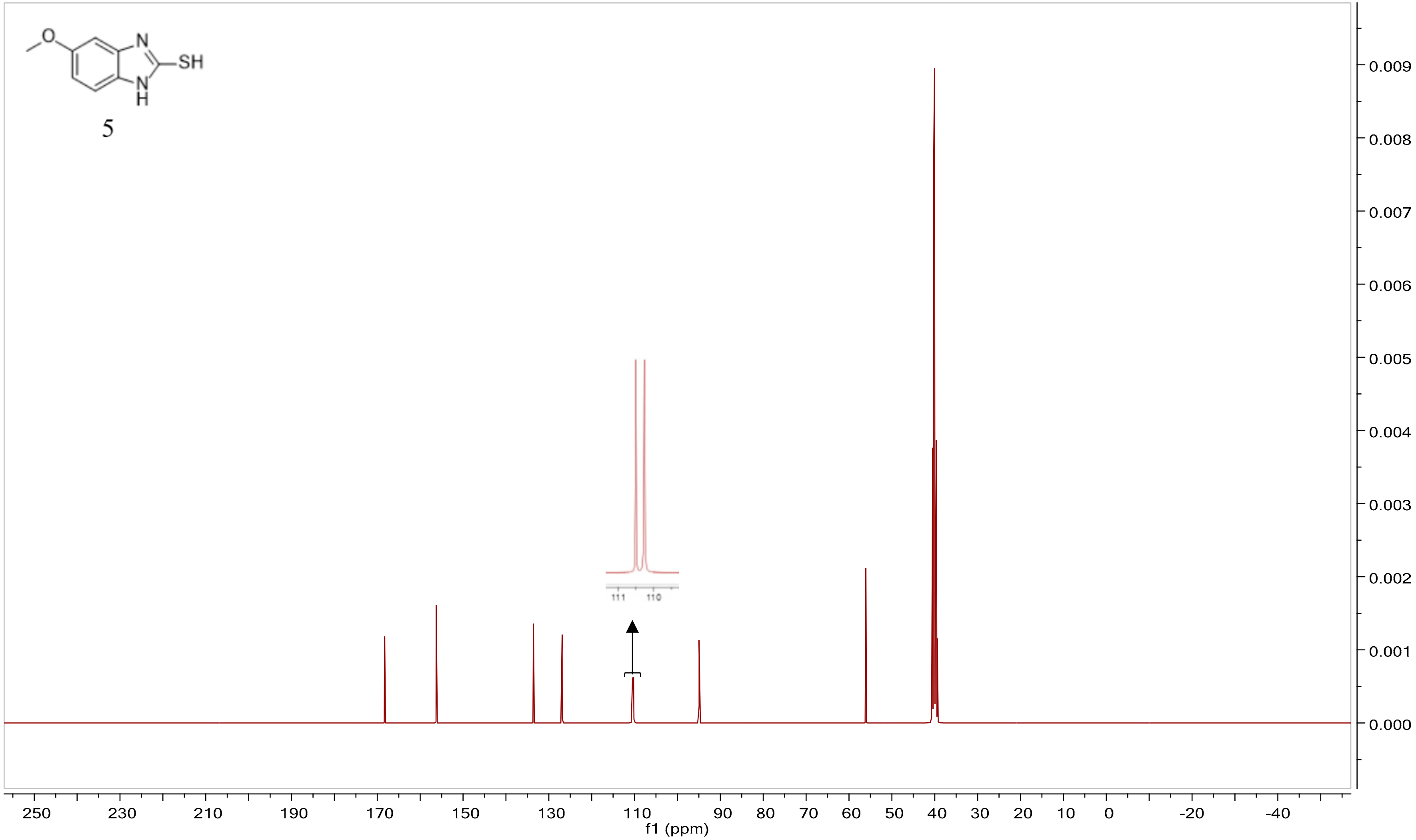

Figure S10. ^{13}C NMR spectrum of analog **5**

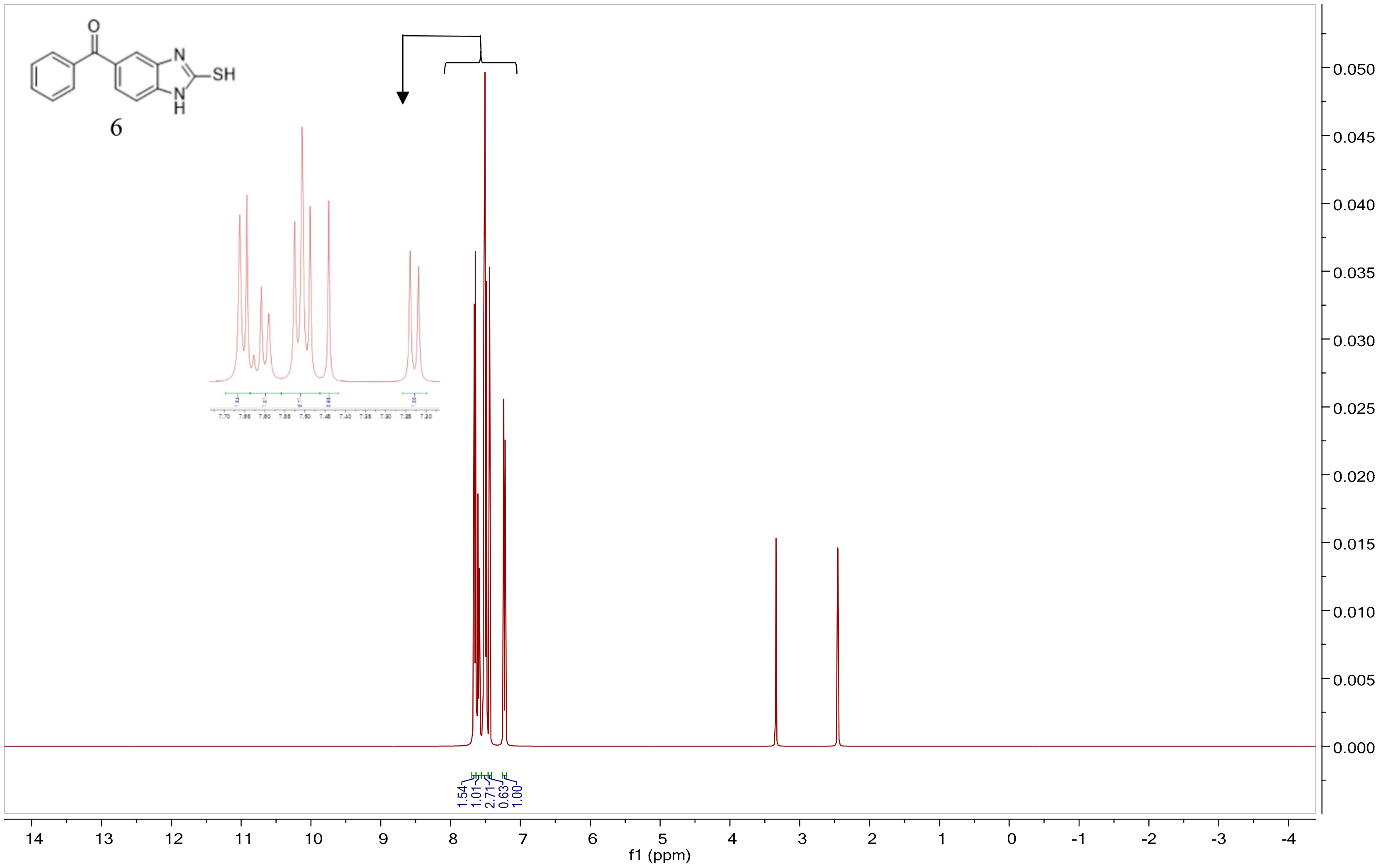

Figure S11. ^{1}H NMR spectrum of analog **6**

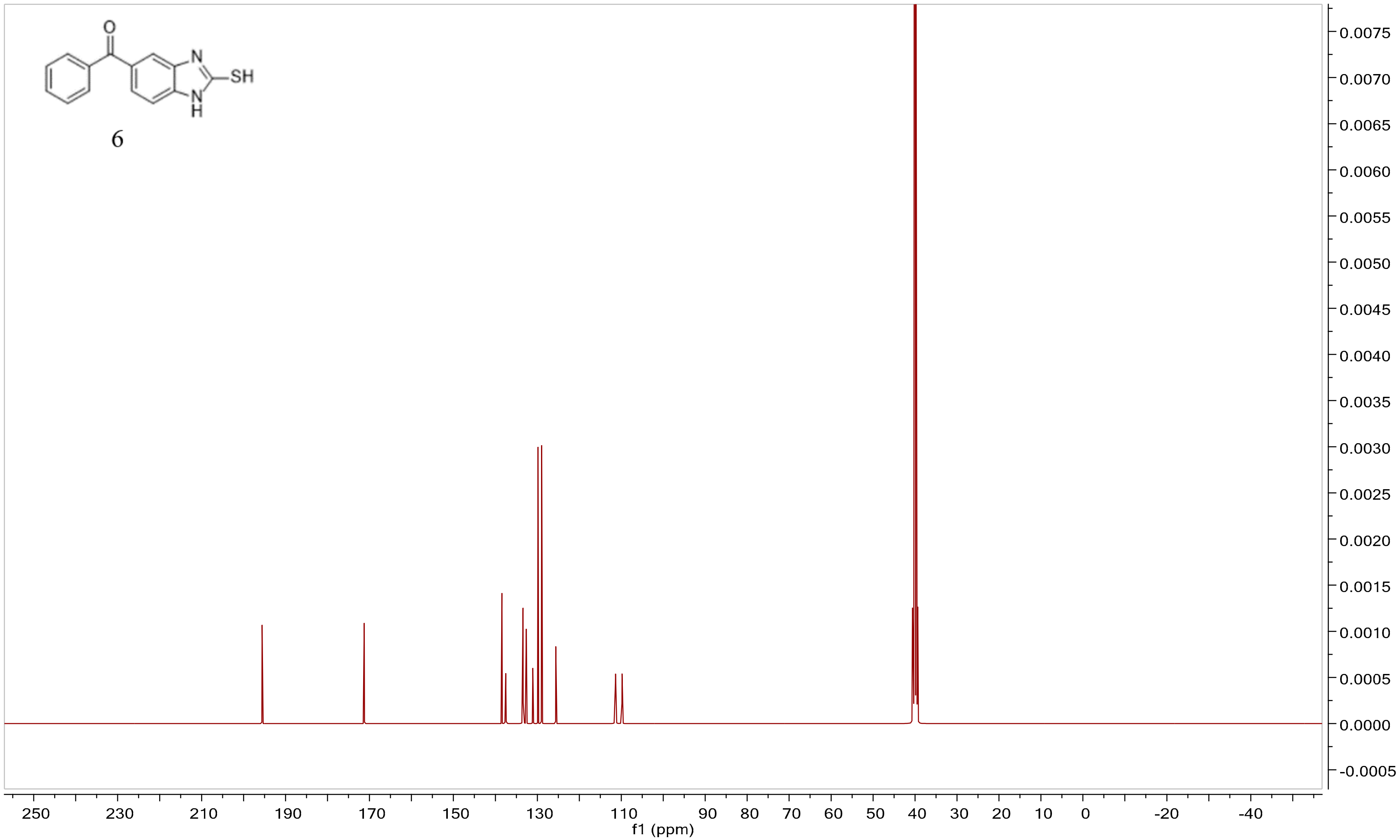

Figure S12. ^{13}C NMR spectrum of analog **6**

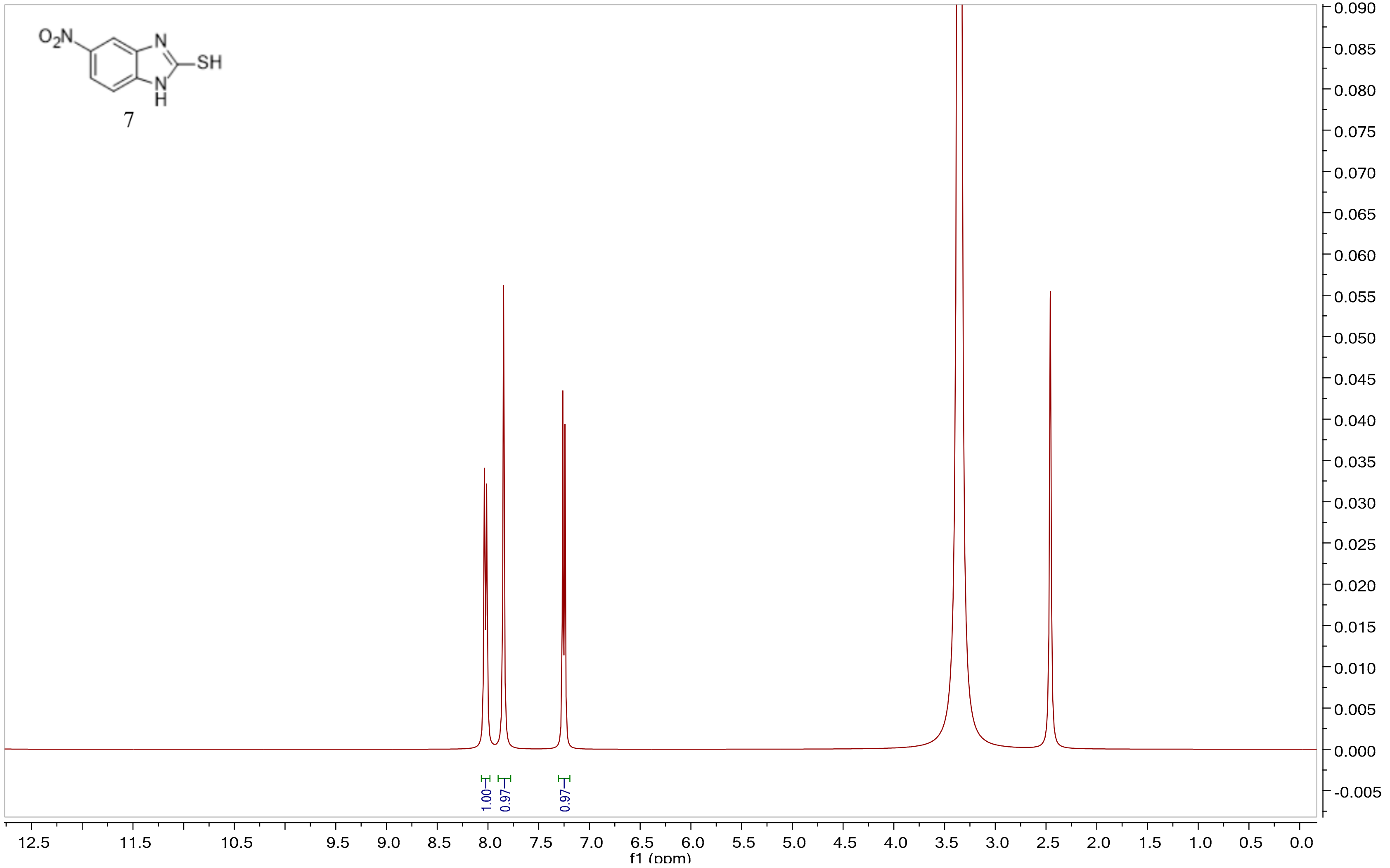

Figure S13. ^{1}H NMR spectrum of analog **7**

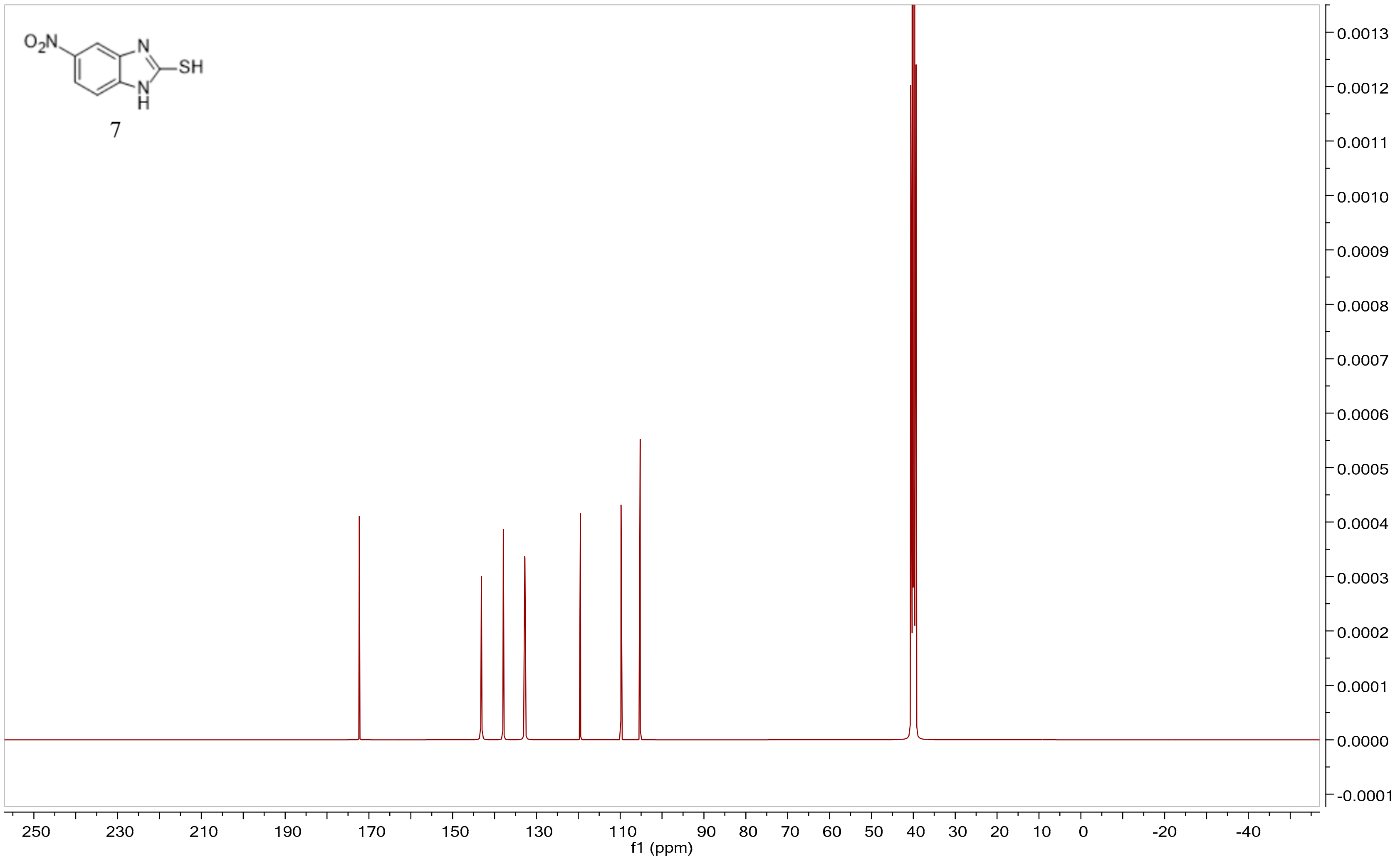

Figure S14. ^{13}C NMR spectrum of analog **7**

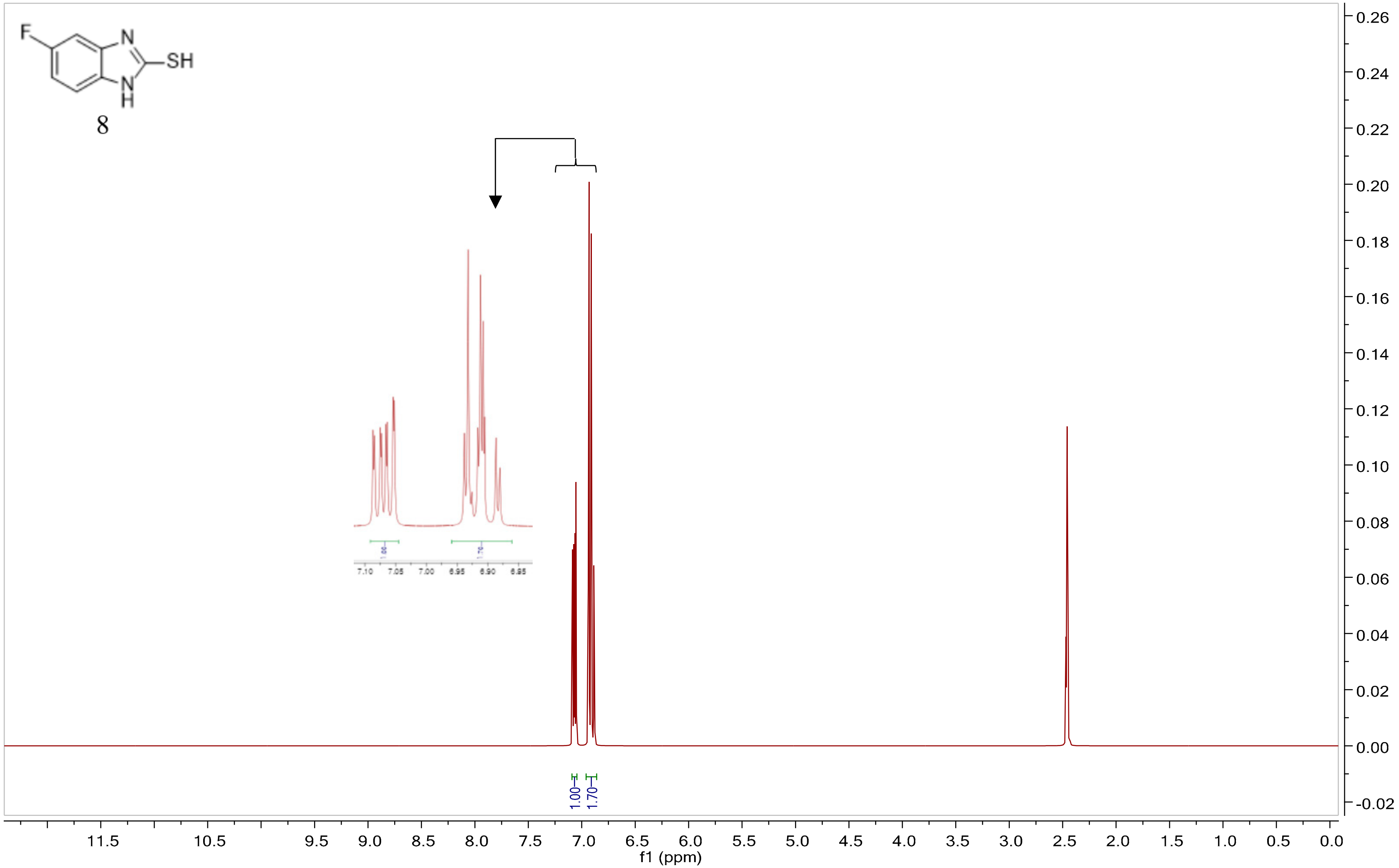

Figure S15. ^{1}H NMR spectrum of analog **8**

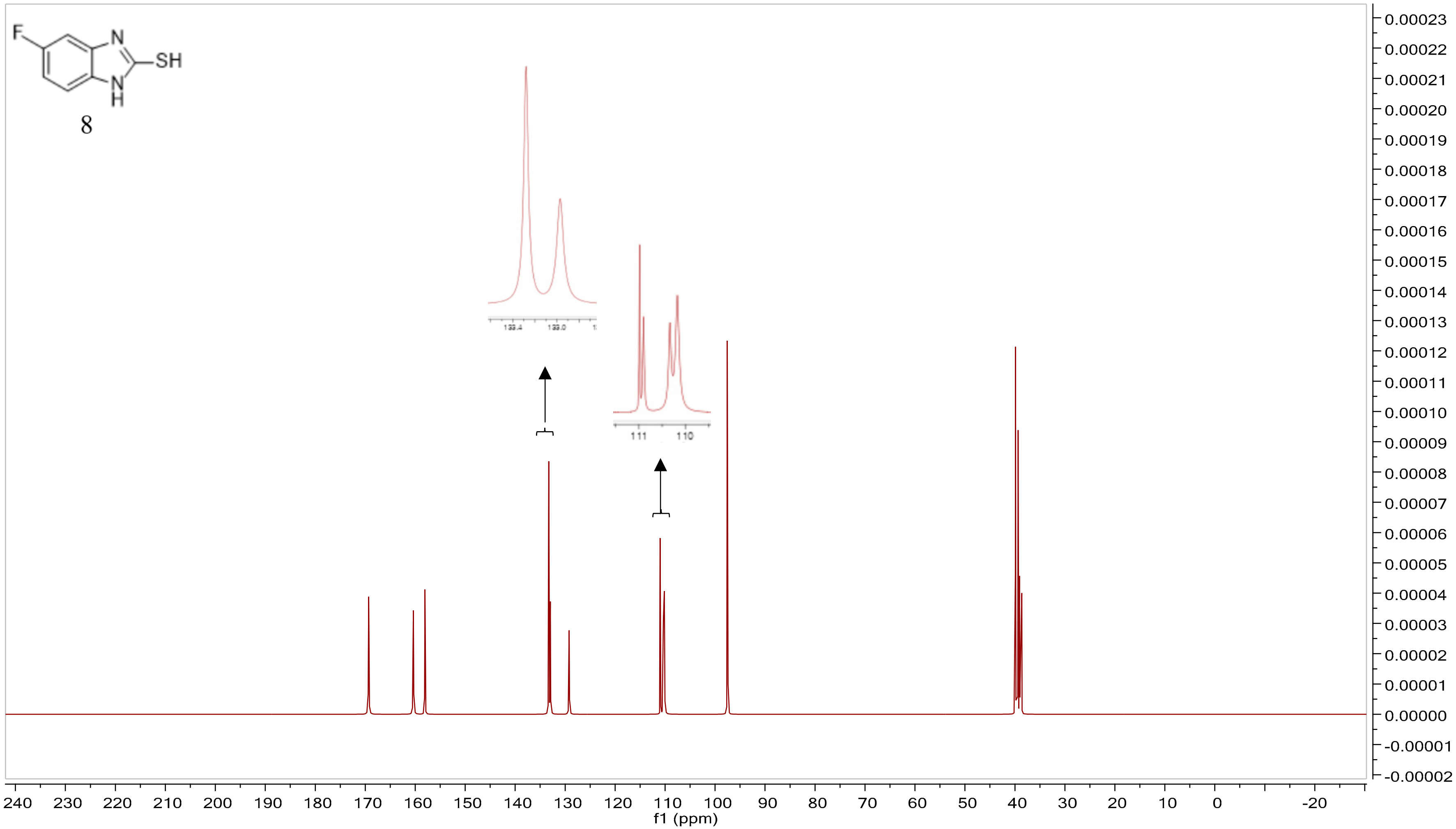

Figure S16. ^{13}C NMR spectrum of analog **8**

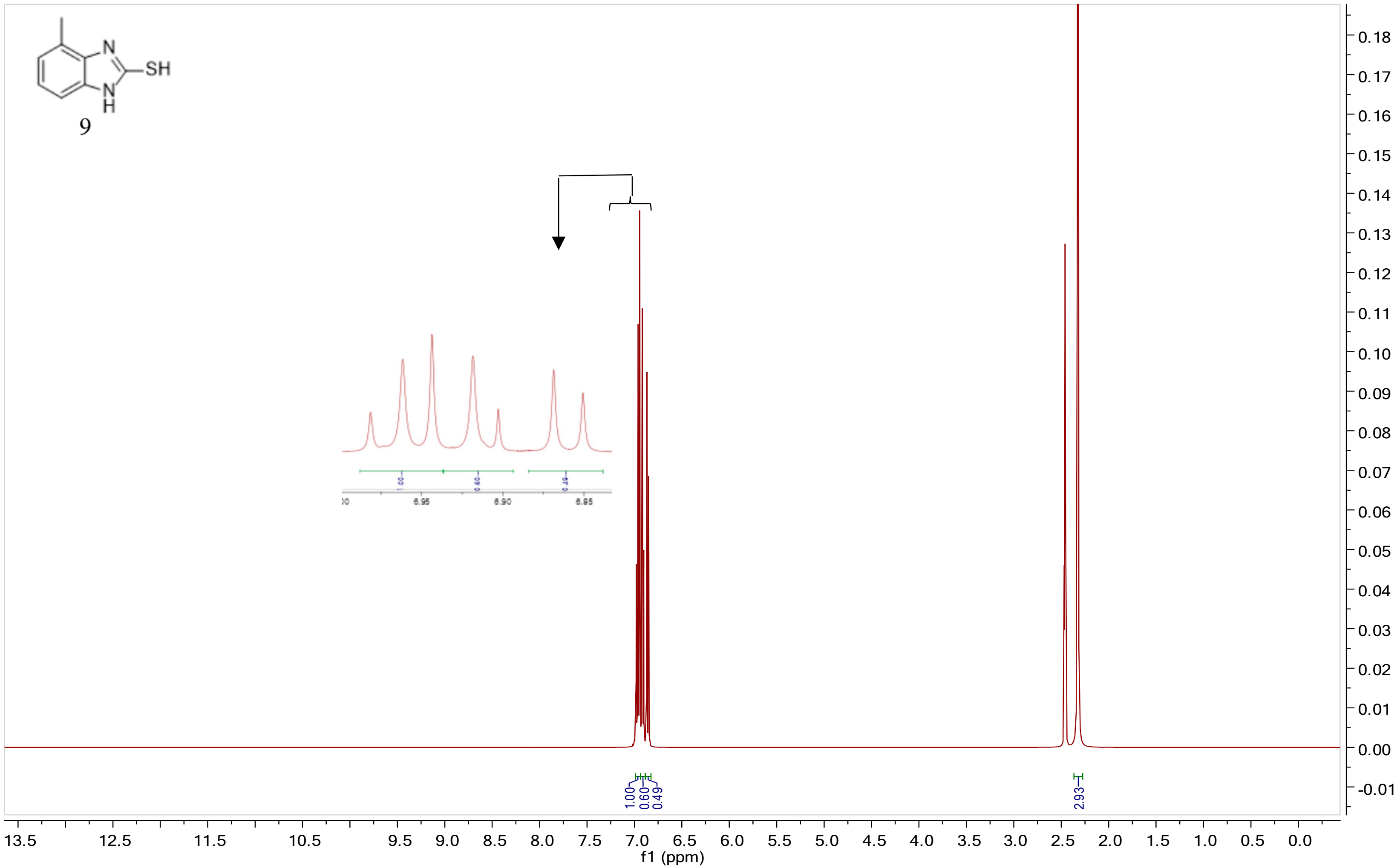

Figure S17. ^{1}H NMR spectrum of analog **9**

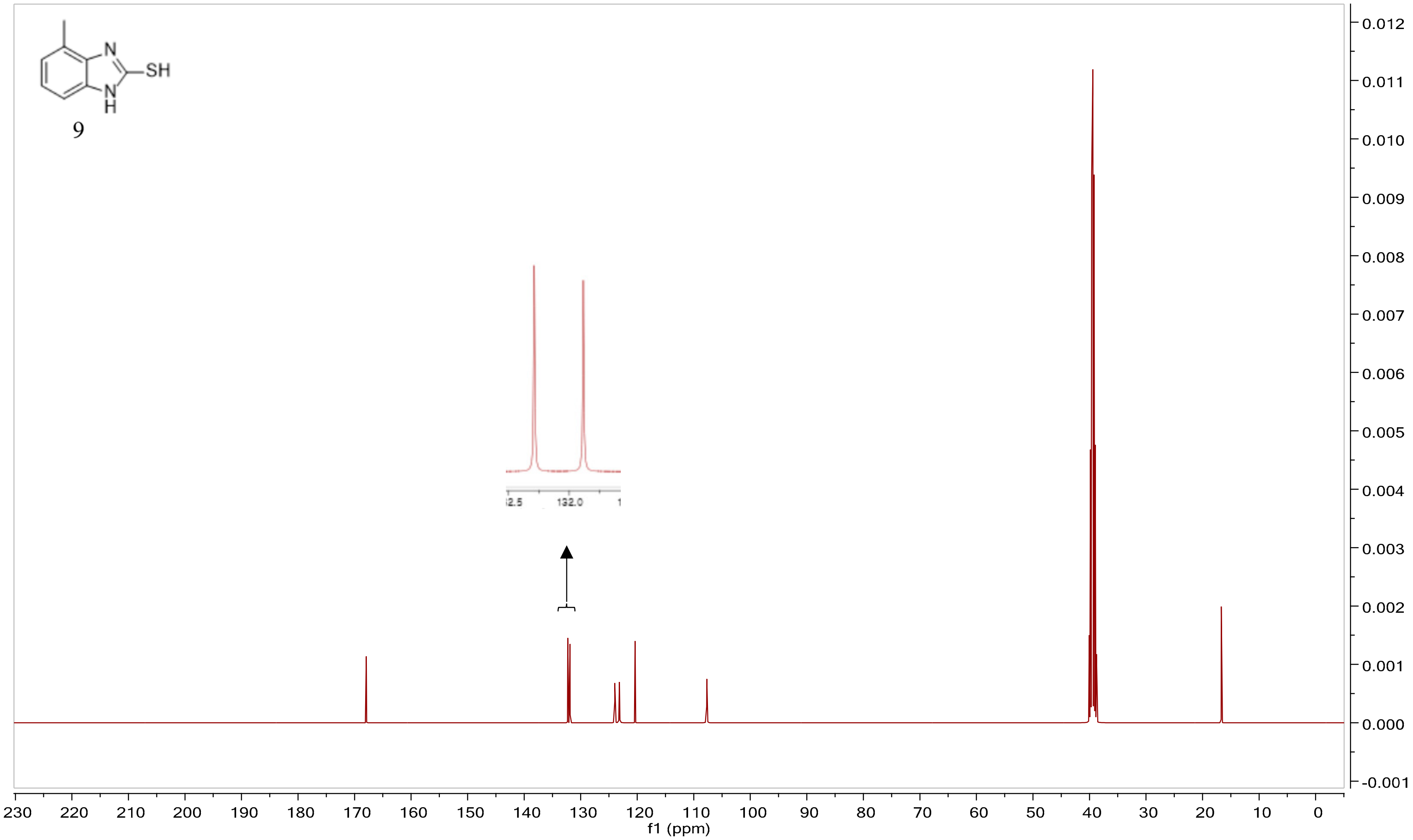

Figure S18. ^{13}C NMR spectrum of analog **9**

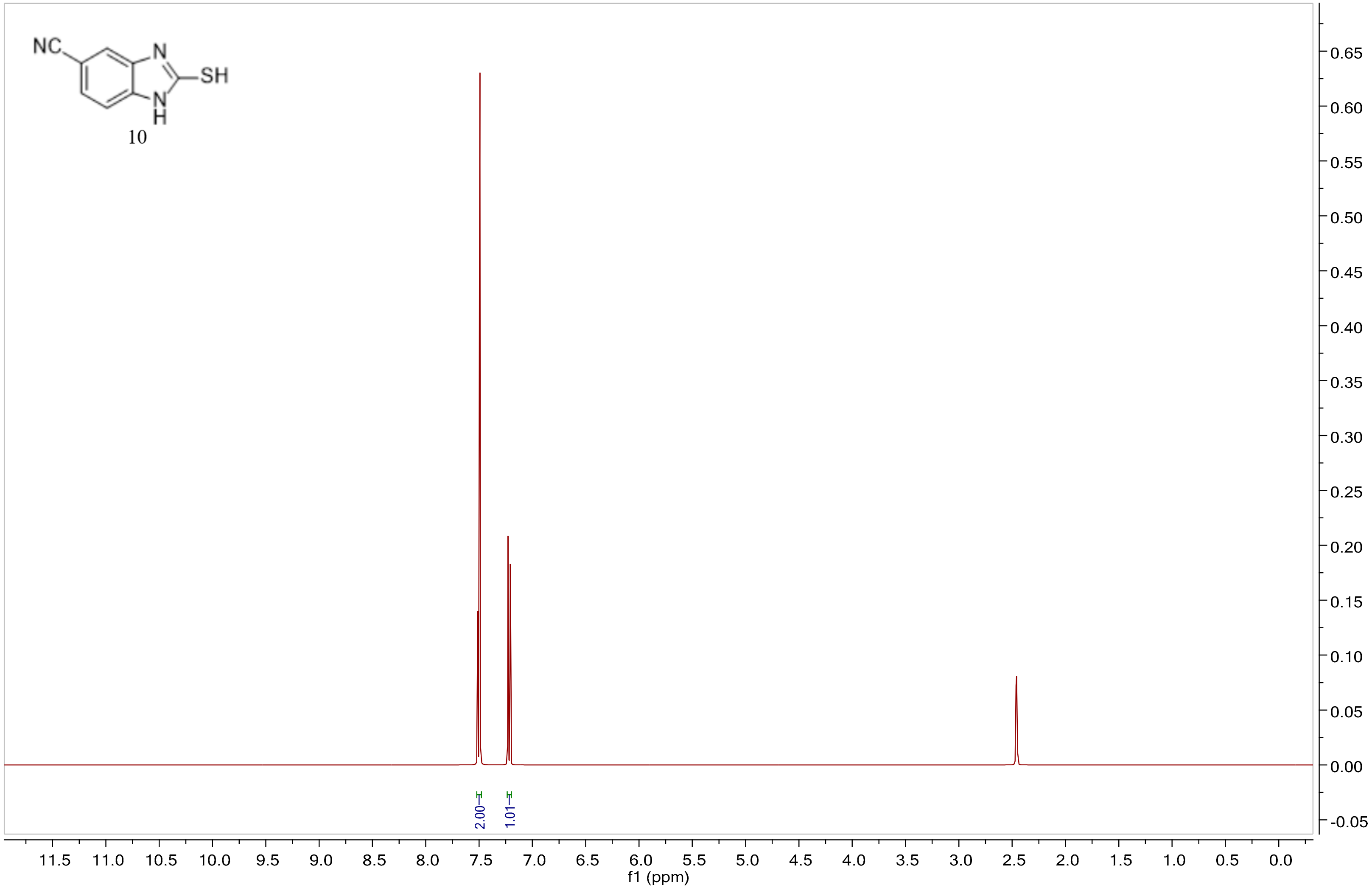

Figure S19. ^{1}H NMR spectrum of analog **10**

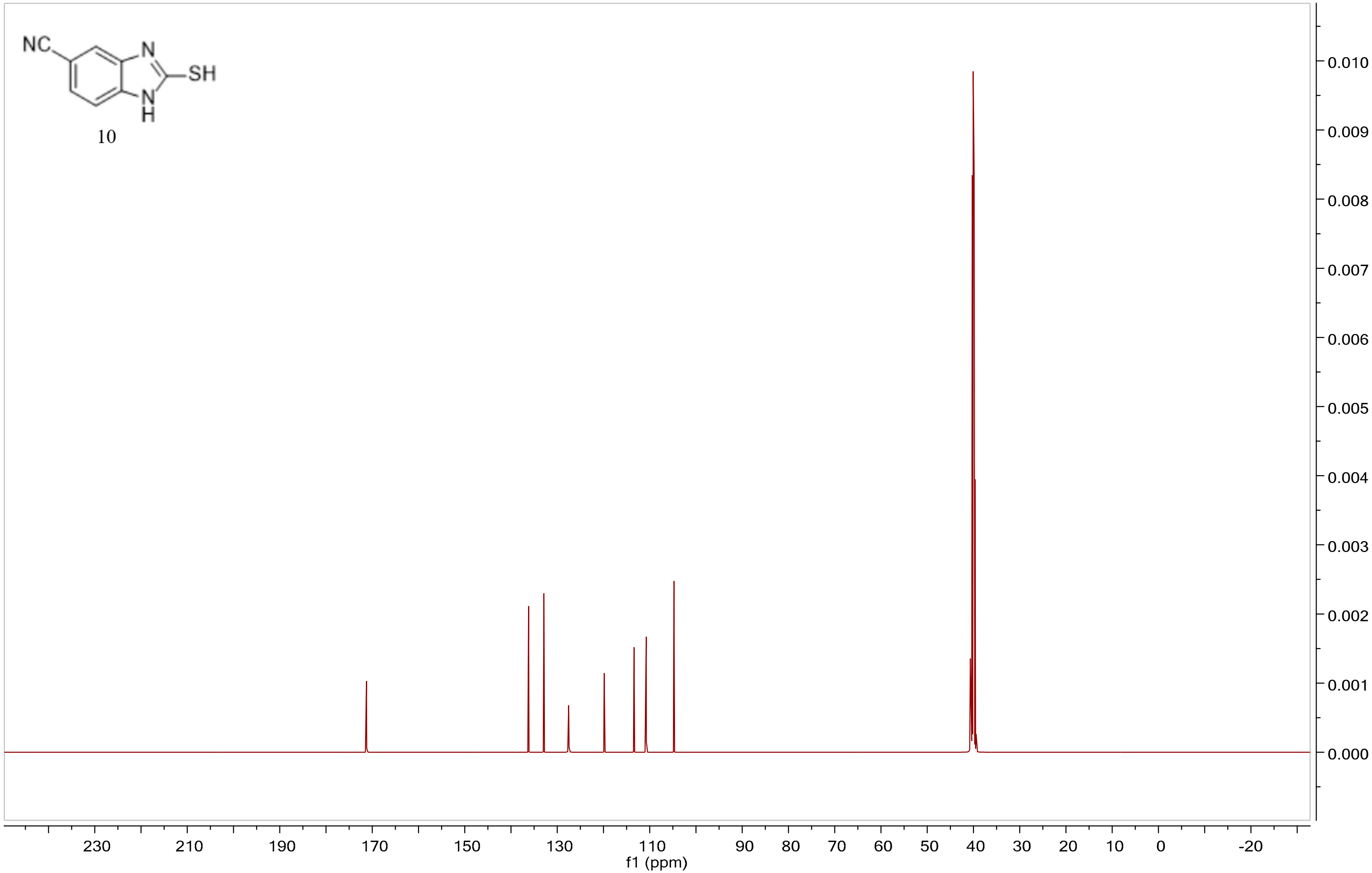

Figure S20. ^{13}C NMR spectrum of analog **10**

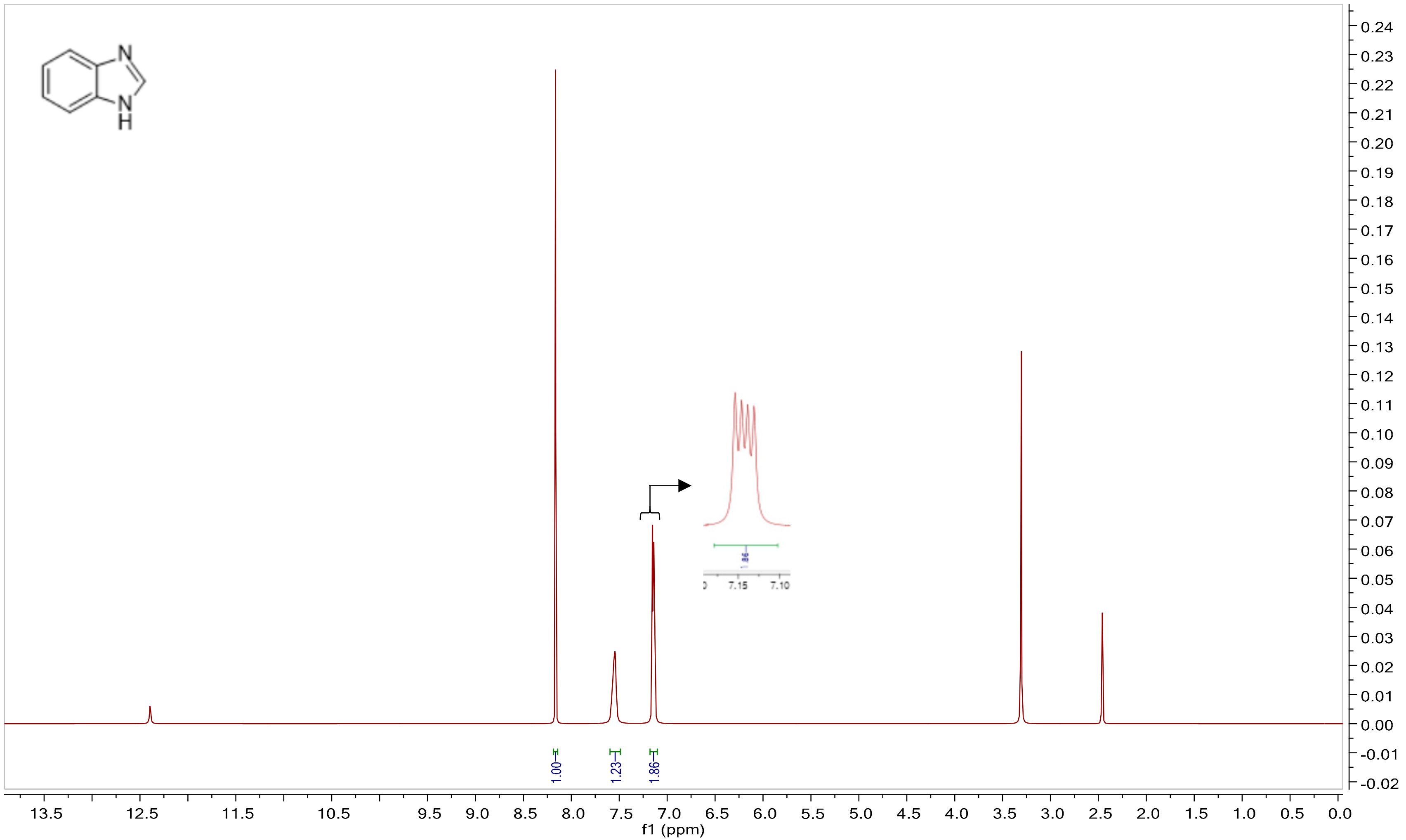

Figure S21. ^{1}H NMR spectrum of analog 1*H*-benzo[*d*]imidazole

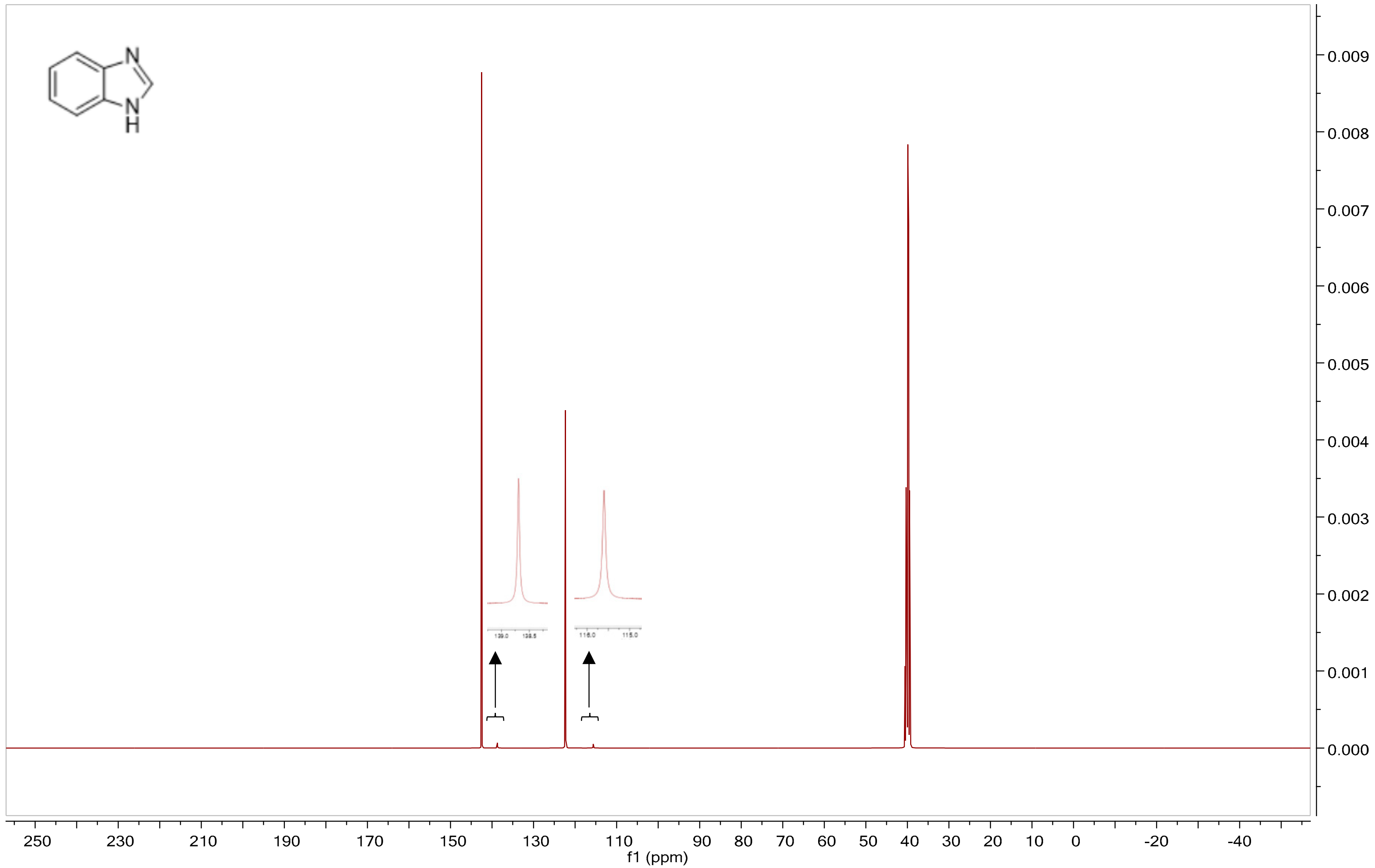

Figure S22. ^{13}C NMR spectrum of analog 1*H*-benzo[*d*]imidazole

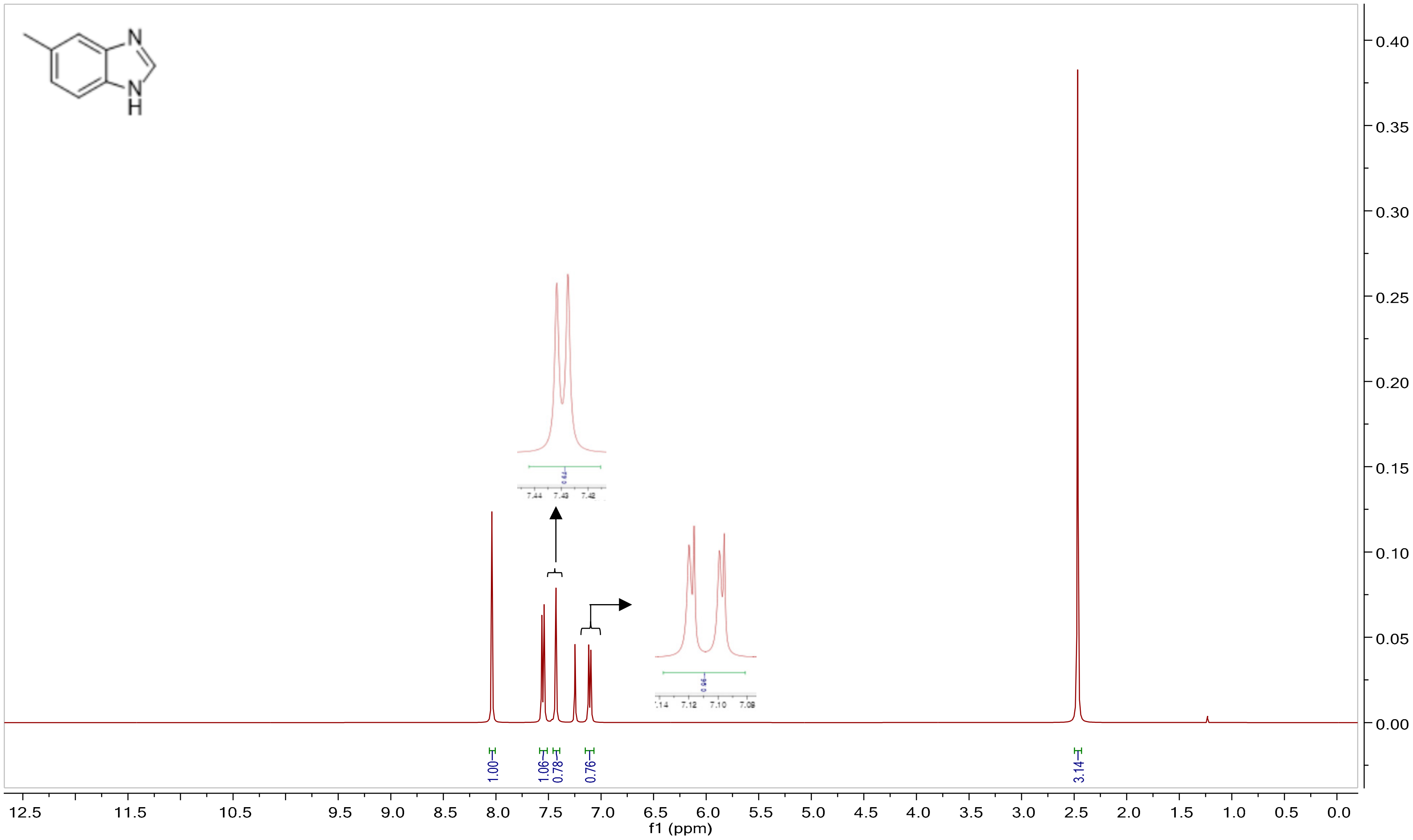

Figure S23. ^{1}H NMR spectrum of analog 5-methyl-1*H*-benzo[*d*]imidazole

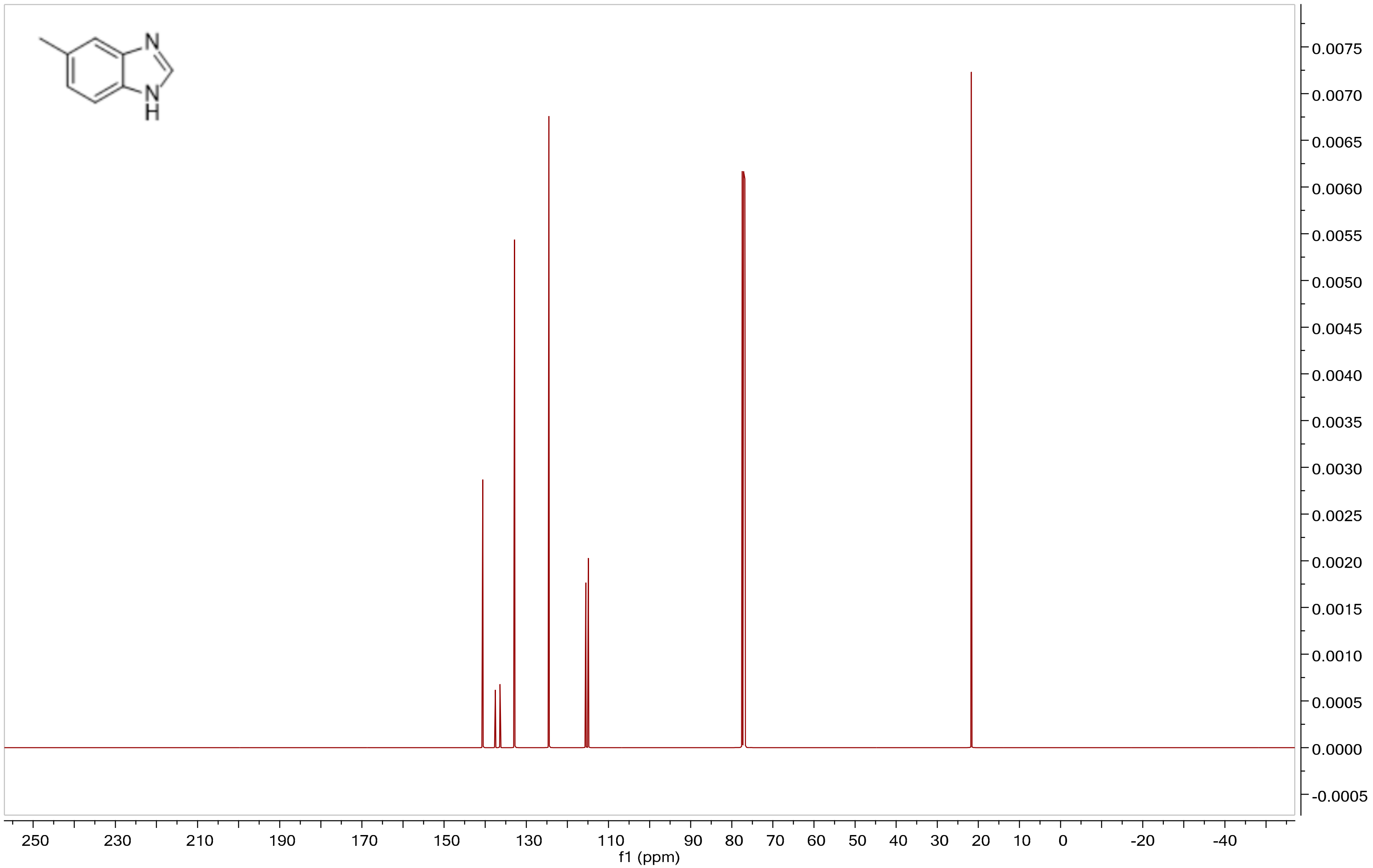

Figure S24. ^{13}C NMR spectrum of analog 5-methyl-1*H*-benzo[*d*]imidazole

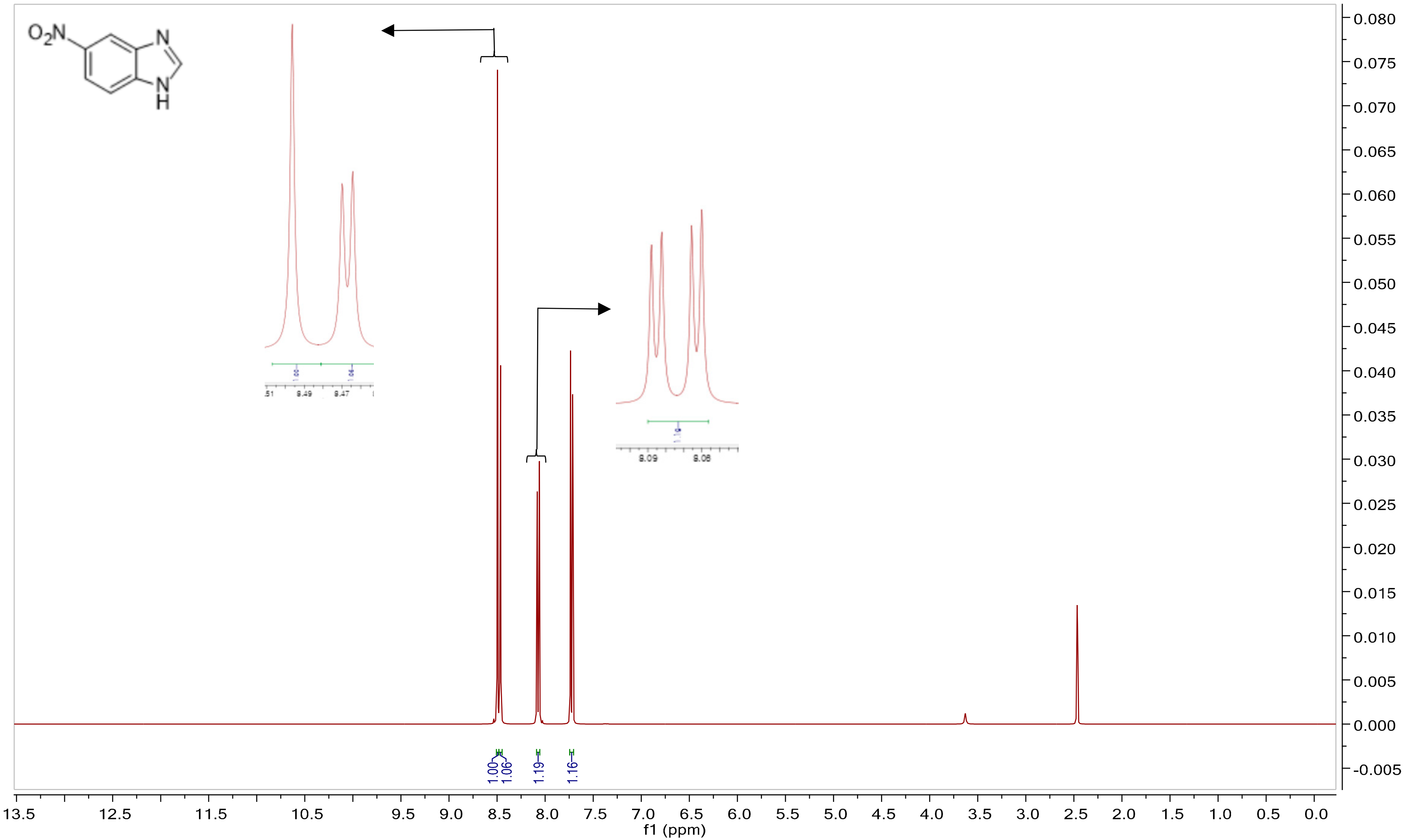

Figure S25. ^{1}H NMR spectrum of analog 5-nitro-1*H*-benzo[*d*]imidazole

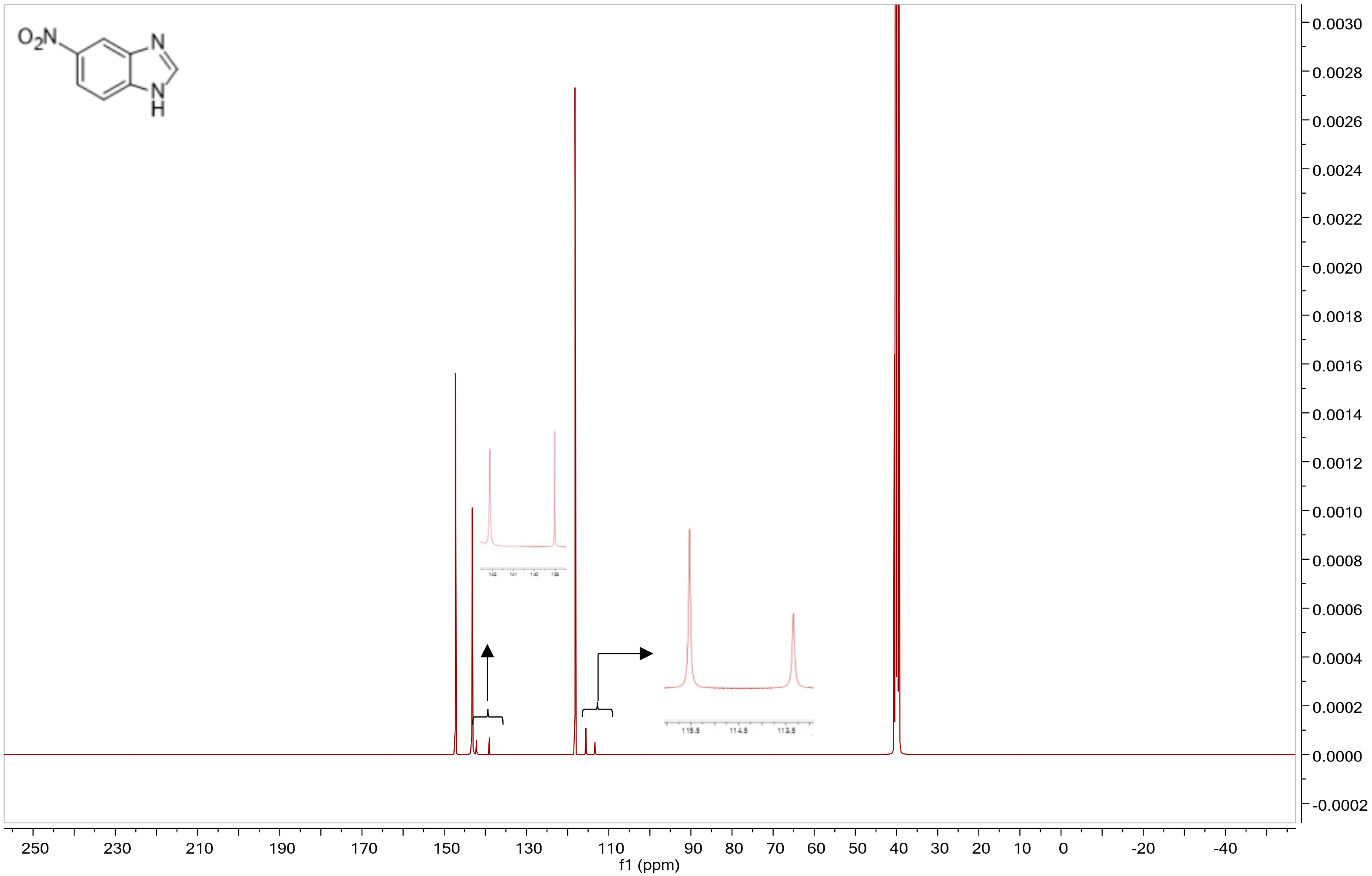

Figure S26. ^{13}C NMR spectrum of analog 5-nitro-1*H*-benzo[*d*]imidazole

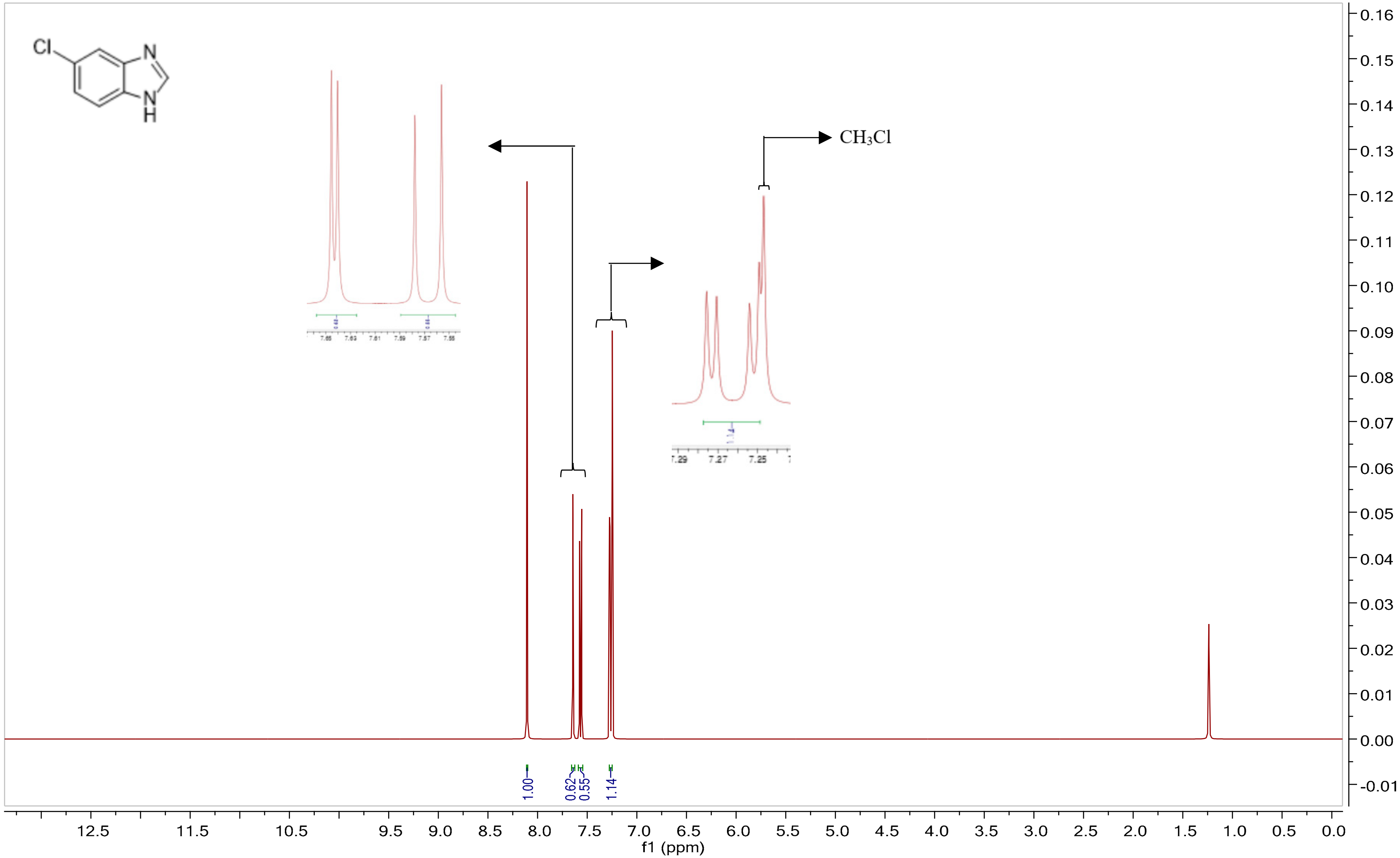

Figure S27. ^{1}H NMR spectrum of analog 5-chloro-1*H*-benzo[*d*]imidazole

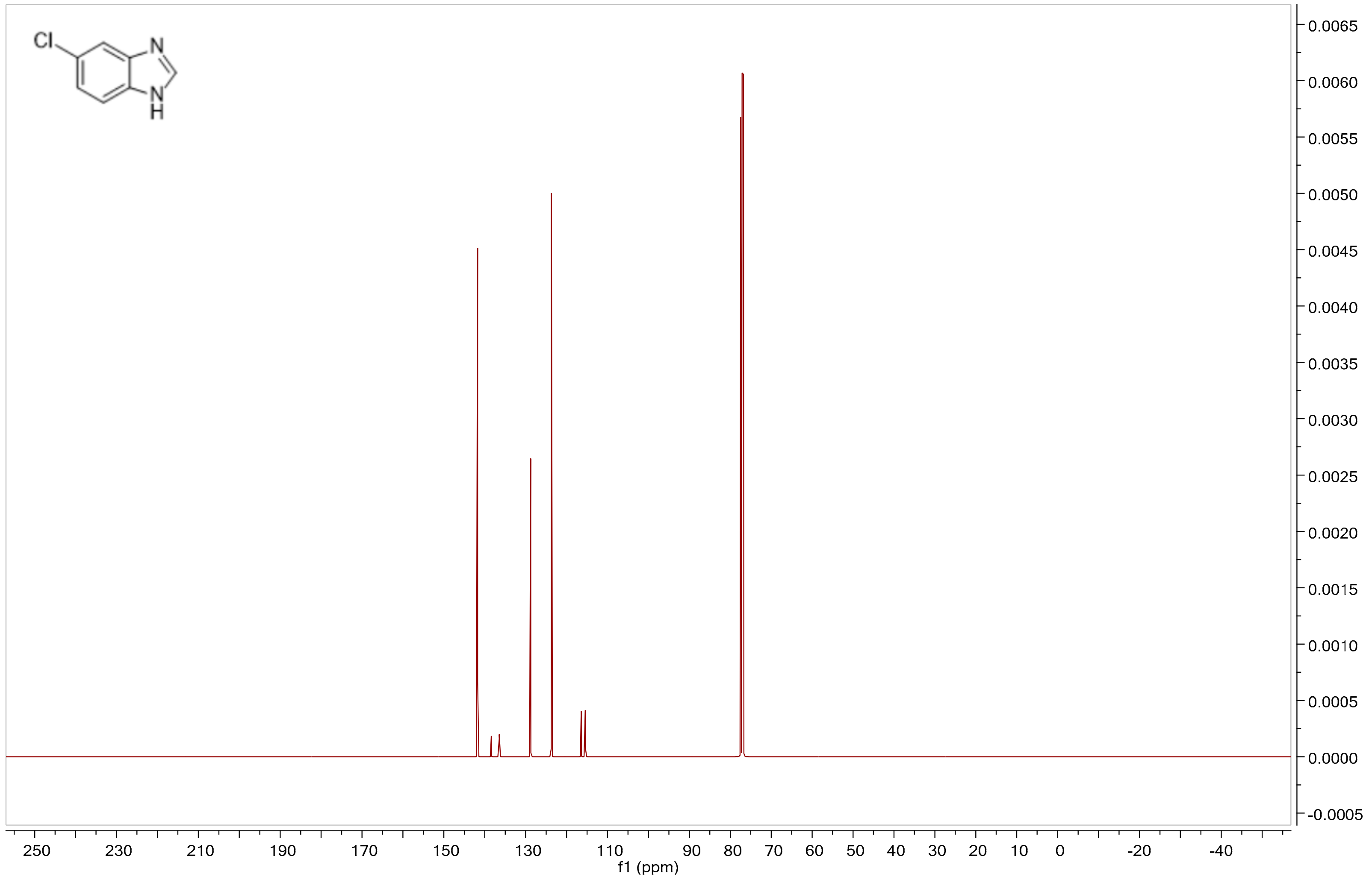

Figure S28. ^{13}C NMR spectrum of analog 5-chloro-1*H*-benzo[*d*]imidazole
